# Microstrain and Crystal Orientation Variation within Naked Triple-Cation Mixed Halide Perovskites under Heat, UV, and Visible Light Exposure

**DOI:** 10.1021/acsenergylett.3c02617

**Published:** 2024-01-08

**Authors:** Yuqin Zou, Johanna Eichhorn, Jiyun Zhang, Fabian A. C. Apfelbeck, Shanshan Yin, Lukas Wolz, Chun-Chao Chen, Ian D. Sharp, Peter Müller-Buschbaum

**Affiliations:** † TUM School of Natural Sciences, Department of Physics, Chair for Functional Materials, Technical University of Munich, James-Franck-Str. 1, 85748 Garching, Germany; ‡ Walter Schottky Institute, Technische Universität München, 85748 Garching, Germany; ■ Physics Department, TUM School of Natural Sciences, Technische Universität München, 85748 Garching, Germany; § Forschungszentrum Jülich GmbH, Helmholtz-Institute Erlangen-Nürnberg (HI ERN), Immerwahrstraße 2, 91058 Erlangen, Germany; ∥ School of Materials Science and Engineering, Shanghai Jiao Tong University, Shanghai 200240, P. R. China; ⊥ Technical University of Munich, Heinz Maier-Leibnitz-Zentrum (MLZ), Lichtenbergstr. 1, 85748 Garching, Germany

## Abstract

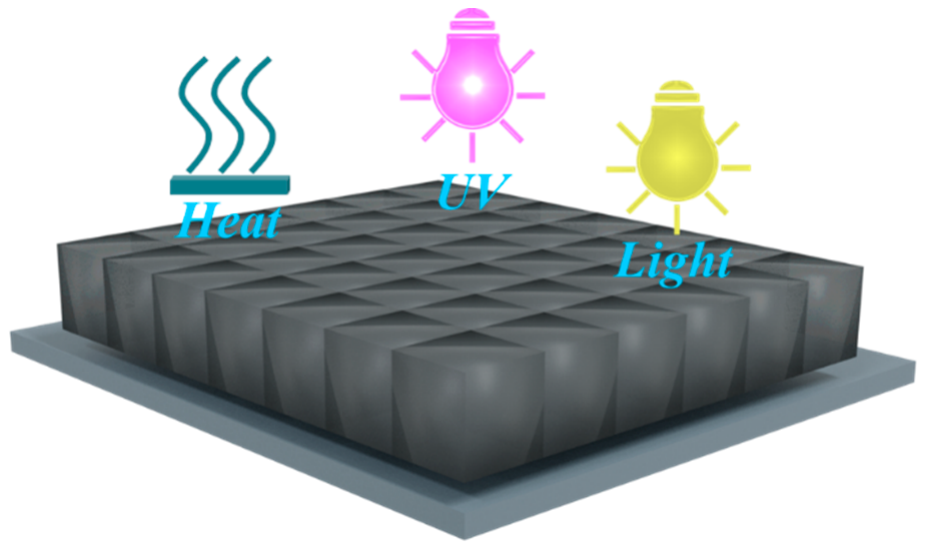

The instability of perovskite absorbers under various environmental stressors is the most significant obstacle to widespread commercialization of perovskite solar cells. Herein, we study the evolution of crystal structure and microstrain present in naked triple-cation mixed CsMAFA-based perovskite films under heat, UV, and visible light (1 Sun) conditions by grazing-incidence wide-angle X-ray scattering (GIWAXS). We find that the microstrain is gradient distributed along the surface normal of the films, decreasing from the upper surface to regions deeper within the film. Moreover, heat, UV, and visible light treatments do not interfere with the crystalline orientations within annealed polycrystalline films. However, when subjected to heat, the naked perovskite films exhibit a rapid component decomposition, induced by phase separation and ion migration. Conversely, under exposure to UV and 1 Sun light soaking, the naked perovskite films undergo a self-optimization structure evolution during degradation and develop into smoother films with reduced surface potential fluctuations.

Solar cell materials based on hybrid organic-lead halide perovskites have attracted significant attention, resulting in a rapid increase in their solar-to-electrical power conversion efficiencies (PCEs). Low-cost solution processing, abundant constituent components, and excellent optoelectronic properties, including remarkable light-absorption coefficient and high defect tolerance, broaden the application prospects of such perovskites, including as photodetectors,^1^ light-emitting diodes,^2^ lasers,^2−4^ and other optoelectronic devices. However, so far the most widespread application is in photovoltaics, with the highest certified PCE of up to 26.1%.^5^ In addition to achieving high efficiencies, a major challenge in the development of perovskite solar cells (PSCs) is to maintain their performance characteristics in harsh environments. In particular, such devices suffer from severe component degradation and phase separation caused by intrinsically detrimental defects and ion migration, as well as extrinsic factors, including oxygen and moisture. Moreover, the degradation of PSCs is further accelerated under operating conditions, in which they are exposed to heat, UV, visible light, and bias voltages.^6−8^ Therefore, an in-depth understanding of the degradation mechanisms associated with poor device stability remains crucial for advancing PSCs toward reliable commercial applications.

Due to its critical importance, the long-term operational stability of PSCs has been extensively investigated. Many studies have tracked the degradation pathways of PSCs operating under exposure to heat, light, and humidity using in situ techniques, including in situ TEM,^6^ nanofocused wide-angle X-ray scattering (nWAXS),^9^ and in situ GIWAXS/GISAXS,^7^ yielding valuable insights regarding perovskite degradation mechanisms. Exposing perovskite materials to these harsh conditions has been found to cause component degradation, ion migration and redistribution within the overall device, and lattice distortions related to microscopic residual strain. Indeed, these effects, as well as their consequences on charge carrier dynamics, photovoltaic performance, and device stability, have been comprehensively explored.^7,10−12^ Many effective strategies have been utilized to hinder or eliminate the degradation routes caused by these stressors.^13−15^ For example, previous work introduced ionic liquid Pyr_14_BF_4_ into the perovskite component to suppress light-induced lattice compression and grain fragmentation, resulting in robust films and improved device stability.^7^

Most studies have thus far focused on degradation of complete devices, which provides a realistic portrait of the degradation suffered by the device under real-world operational conditions. However, this approach makes it difficult to unambiguously distinguish the specific contributions of interfaces, charge transport layers, and electrodes to the instability. Consequently, degradation of the active layer itself can be obscured and may not be accurately probed. Moreover, it is difficult to ensure that insights gained from optimization of the complete device stability can be applied to other systems comprising different perovskite components and device structures. To overcome these limitations, we investigate the degradation processes within naked perovskite thin films upon exposure to heat, UV, and visible light, which reveal a stable distribution of crystal orientations but modification of the surface elemental distribution associated with ion migration and decomposition, as well as changes to the surface potential. In particular, we systematically monitor the variation in morphology and crystal structure (crystal phase, crystal orientation, and microscopic residual strain) of naked triple-cation mixed halide perovskite (Cs_0.05_(FA_0.85_MA_0.15_)_0.95_Pb(I_0.85_Br_0.15_)_3_, denoted as CsMAFA) thin films under each of these environmental stimuli. Grazing-incidence wide-angle X-ray scattering (GIWAXS) is used to precisely probe the crystallite structure and preferential crystallite orientation, while also enabling in situ tracking of microscopic residual strain variations within films at different probing depths.^16^ Complementary ex situ X-ray photoelectron spectroscopy (XPS) provides key information regarding the composition of the film surface and is used to track ion migration and component degradation under environmental stressors. Using these methods, along with scanning probe microscopy, we comprehensively analyze these heat-, UV-, and visible light-induced changes in naked CsMAFA films, as well as their effects on the film surface roughness and the lateral distribution of surface potentials. Thus, we provide new insights that can guide the design of practical approaches to stabilization that can be applied to various device structures based on conventional (n-i-p) and inverted (p-i-n) PSC architectures alike, as well as to flexible or tandem devices.

## Characterization Techniques, Device Structure, and Photovoltaic Performance

**Figure 1:**
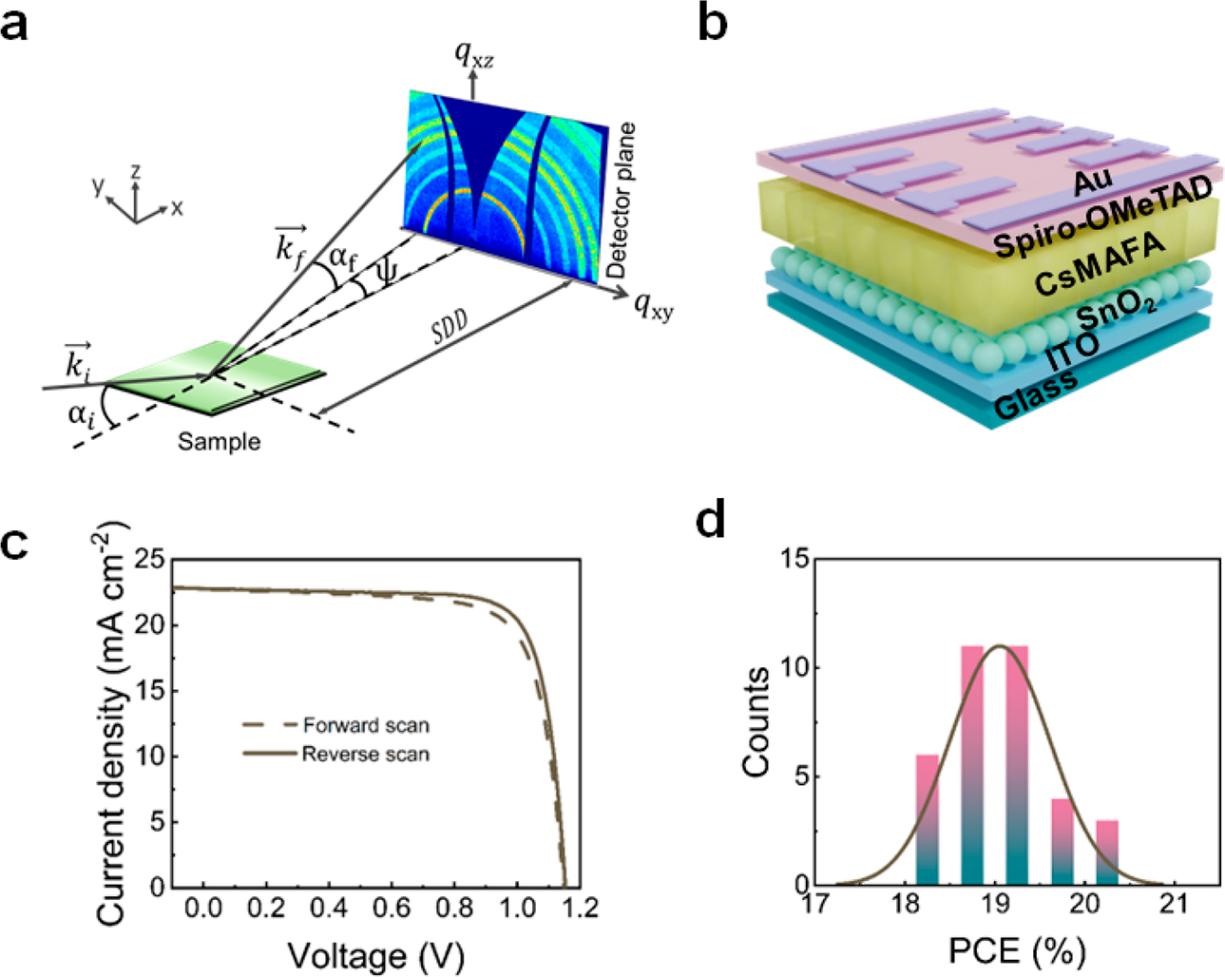
(a) Schematic drawing of the GIWAXS setup; the diffuse scattering signal is collected with a 2D detector. The sample surface is inclined by an incident angle α_i_ against the horizon. The exit angle is denoted α_f_, and the out-of-plane angle is ψ. The color-coding visualizes differences in the scattered intensity. The sample–detector distance (SDD) for GIWAXS is set to ∼0.1 m. (b) Schematic of the n-i-p perovskite solar cell: ITO/SnO_2_/CsMAFA/spiro-OMeTAD/Au. (c) *J*–*V* curves of the champion device, with forward and reverse scan measured under 1 Sun (100 mW cm^–2^) illumination. (d) Statistical distribution of PCE values obtained from *J*–*V* characteristics of 35 cells.

Figure 1a shows a schematic overview of the GIWAXS experimental setup, where the *x*-axis is oriented along the X-ray beam direction, the *y*-axis is parallel to the sample surface, and the *z*-axis is along the surface normal.^17^ All angles are probed with respect to the film surface, and the GIWAXS signal is collected with a 2D detector. From GIWAXS measurements, we obtain information about the crystallite structure by probing reciprocal lattice distances and about the preferential crystallite orientation with respect to the substrate surface by analyzing the azimuthal intensity distribution of the scattering intensity along the specified Bragg ring. Detailed information about the GIWAXS measurements, including corrections, radial cuts, and azimuthal tube cuts, can be found in the Supporting Information. More information about the general GIWAXS analysis can be found in the literature.^17^

In the present study, we select CsMAFA as the perovskite absorber layer due to its high device efficiency and widespread application in the perovskite photovoltaics field. To confirm that the CsMAFA perovskite thin films studied here function well as the active medium in solar cells, we first prepare complete devices with a n-i-p architecture (indium tin oxide (ITO)/SnO_2_/perovskite/spiro-OMeTAD/Au). The device structure is shown in Figure 1b, and details regarding the device architecture, perovskite precursor solution composition, and fabrication procedure are described in the Supporting Information. Figure 1c shows the current density–voltage (*J*–*V*) curves of the champion device in the forward and reverse scan directions. The obtained PCE is 20.47%, with a short-circuit current density (*J*_SC_) of 22.78 mA cm^–2^, an open-circuit voltage (*V*_OC_) of 1.15 V, and a fill factor (FF) of up to 0.78. Figure 1d and Table S1 summarize the statistical distribution of PCEs and detailed parameters extracted from 35 devices, respectively. The other photovoltaic parameter distributions are plotted in Figure S1. The average PCE is 19.06%, with an average *J*_SC_ of 21.51 mA cm^–2^, an average *V*_OC_ of 1.15 V, and an average FF of 0.77. Here, three distinct stress conditions are selected to investigate the degradation of CsMAFA perovskite films: heated at 150 °C (labeled heat), UV light illumination with a wavelength of 395–400 nm (labeled UV), and 1 Sun illumination (standard AM 1.5G, labeled visible light). All experiments were conducted in a N_2_-filled glovebox, with the O_2_ value maintained about 2 ppm, H_2_O value below 0.1 ppm, thereby minimizing the influence from other environmental factors.

## Morphology and Crystal Structure Variation of CsMAFA Films under Heating Conditions

**Figure 2:**
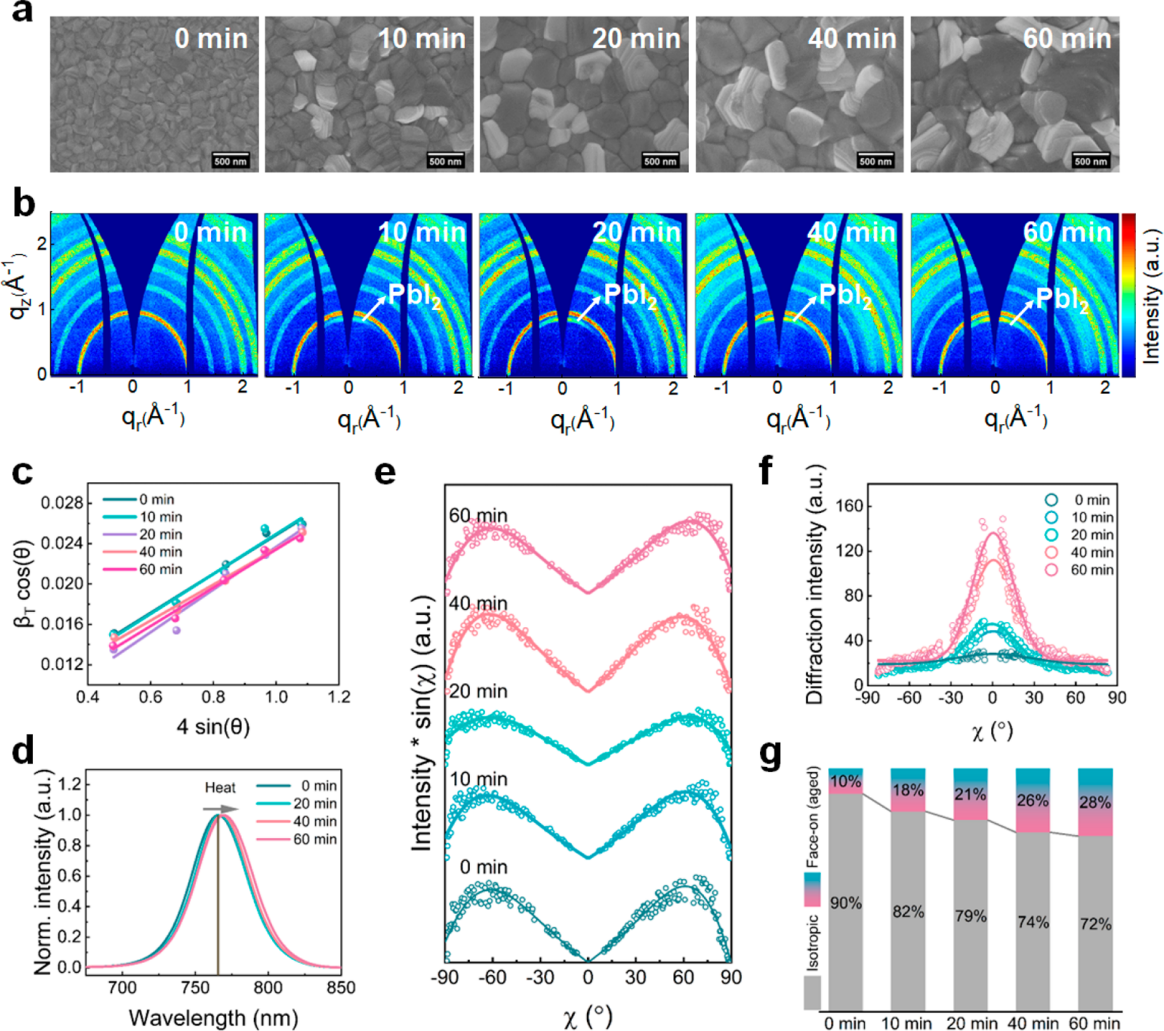
CsMAFA perovskite films heated at 150 °C for different times. (a) Top-view SEM images, in which the scale bar is 500 nm. (b) 2D GIWAXS data measured at incident angle α_i_ = 0.4°. (c) Calculation of the microstrain distribution, obtained via linear fit of β_T_ cos θ vs 4sin θ. (d) PL spectra. (e) Azimuthal tube cuts along the (002/110) Bragg peak at around *q* = 1.00 Å^–1^ from the 2D GIWAXS data in panel b measured at α_i_ = 0.4°. (f) Azimuthal tube cuts along the PbI_2_ Bragg peak at around *q* = 0.90 Å^–1^ from the 2D GIWAXS data in panel b measured at α_i_ = 0.4°. (g) The relative material quantity (MQ) of each orientation extracted from panel f.

We first explore the morphology and crystal structure variations of CsMAFA perovskite films upon heating at 150 °C as a function of time. Figures 2a and S2 show the time-dependent top-view scanning electron microscopy (SEM) images with scale bars of 500 nm and 2 μm, respectively, which provide insights into the changing microscale morphology of the CsMAFA films during annealing at 150 °C. As the heating time increases, the small and well-defined grains begin to grow and coalesce to form larger crystal domains by consuming smaller neighboring grains. This morphological change is accompanied by the generation of a substantial amount of PbI_2_, as indicated by the emergence of a white phase that is clearly visible in the heated films.^18^ The generation of PbI_2_ phase is further confirmed by the time-dependent ex situ 2D GIWAXS data (transformed to *q*-space) of the CsMAFA film annealed at 150 °C shown in Figure 2b. For these measurements, the incidence angle, α_i_, is fixed at 0.4°. As the heating time increases, we observe an increasing intensity of the Bragg ring at 0.90 Å^–1^ associated with PbI_2_, indicating the continuous generation of the PbI_2_ phase. To visualize this thermally induced phase reconfiguration, radial cake cuts (azimuthal integration from −90° to 90° in χ direction) are performed, as shown in Figure S3, yielding data comparable to conventional X-ray diffraction (XRD) and, thus, named pseudo-XRD cuts. The Bragg peaks located at approximately 1.00 Å^–1^, 2.00 Å^–1^, and 2.20 Å^–1^ are assigned to the (110), (220), and (310) planes of the CsMAFA perovskite phase, respectively. The emergence of the Bragg peak at around 0.90 Å^–1^ indicates the formation of the PbI_2_ phase, corresponding to the Bragg ring of PbI_2_ from the 2D GIWAXS data (Figure 2b) and in agreement with SEM results (Figure 2a).

We further use the Williamson–Hall analysis for azimuthally integrated line profiles to track the microstrain evolution in naked CsMAFA films as a function of heating time.^19^ The Williamson–Hall method relies on the different *q*-dependence of Bragg peak broadening resulting from microstrain and crystallite size effects.^12^ The diffraction data are fitted with a Gaussian distribution function to obtain the peak positions and full width at half maximum (fwhm) values (Figure S4). Information regarding the detailed data treatment can be found in Supplementary Note 1 and Table S2. Generally, the quantity β_T_ cos θ follows a linear relationship with 4sin θ, with the slope of the fit lines representing the magnitudes of the microstrain (see eq 5 in Supplementary Note 1). As shown in Figure 2c and Table S2, the microstrain values (from 0.020 ± 0.002 to 0.019 ± 0.001) remain consistently similar across all CsMAFA films heated for different times, indicating a weak correlation between the microstrain levels in naked CsMAFA films and the heating duration (when the incidence angle α_i_ is fixed at 0.4°). In earlier work, it was found that the microscopic residual strain was correlated to the local lattice distortion caused by compositional heterogeneity and was accompanied by phase separation of the mixed perovskite.^10,12,20^ A new phase will form when there is a separation into regions with distinct compositions and lattice parameters. However, slight redistribution of ions would lead to a slight change in the distribution of lattice constants rather than an entirely new phase, which explains why we do not observe new Bragg peaks in the scattering data but we observe a slight shift in the emission energy of photoluminescence (PL) spectra (Figure 2d).

PL measurements provide further insight into thermally induced changes of the perovskite films and are sensitive to subtle changes of the bandgap that can occur due to phase separation or ion redistribution. Figure 2d shows the normalized PL spectra of CsMAFA films heated at 150 °C for different times. The PL fit data and fit parameters (analyzed with a Gaussian function and then normalized) are shown in Figure S5 and Table S3, respectively. A redshift positively correlated with the heating time is observed for the PL peak, which shifts from 766.38 ± 0.04 nm to 770.20 ± 0.03 nm. Since the emission photon energy is determined by low bandgap regions of mixed phase material, such a red-shift may be related to redistribution of ions within the films. In particular, this behavior is consistent with a relative enrichment of FA^+^ (large cation, compared with MA^+^ and Cs^+^) on the A-site of heated perovskites, since FA-rich perovskites have larger lattice constants and smaller bandgaps (FAPbI_3_, ∼1.48 eV^21^).^22,23^ At elevated temperature, the volatile MAI mainly distributed on the film surface suffers more severe losses because MA is less thermally stable than FA.^10^ Another possible reason for this behavior could be halide ion migration and spatial redistribution upon heating, resulting in I-rich and Br-rich crystalline domains from the original stoichiometric mixed cation and halide perovskite phase.^22−25^ We speculate that the phase segregation could be driven by the heat-induced local structural microstrain changes, as discussed below in the context of XPS analysis.

Next, we perform azimuthal tube cuts along the (110)/(002) Bragg peaks from the 2D GIWAXS data to gain insight into the distribution of crystallite orientations within the films. The azimuthal tube cut data and Gaussian profile fits are shown in Figure S6. The Bragg peak is weak and broadly distributed over the azimuthal angular range, suggesting the lack of pronounced film texture and random crystallite distribution with this orientation. Figure 2e shows the pole figures, in which the intensity is Lorentz-corrected by multiplication with sin χ, reflecting the intensity distribution of the relative materials quantity (MQ), which is defined as the fraction of preferentially oriented crystallites within the total number of crystallites (MQ_rel_ = MQ/MQ_tot_, with MQ_tot_ = ΣMQ, of the respective orientation: face-on, edge-on, and isotropic^26^). All films show two Gaussian-shaped peaks at χ ≈ ±60°, indicating an edge-on orientation, in which the (110)/(002) planes of the perovskite phase are tilted by 60° with respect to the substrate surface. The occurrence of the edge-on orientation shows very little correlation with the heating time since almost all fits are similar. However, the PbI_2_ phase (Figure 2f) shows a distinct orientation around χ ≈ 0°, which corresponds to the (001) plane of PbI_2_ crystals being oriented parallel to the substrate surface. The fraction of the face-on and isotropically oriented crystallites is shown in Figure 2g, estimated by integrating the areas under the corresponding features in the pole figures (Lorentz corrected intensity × sin(χ) versus χ plots seen in Figure S7). The face-on and isotropic MQ are marked with red-green and gray shaded regions, respectively. For the (001) plane of PbI_2_ phase, crystallites with isotropic orientation dominate in all samples. With increasing the annealing time, the face-on oriented MQ increases from 10% to 28%, indicating that the newly generated PbI_2_ crystallites are predominantly stacked along the face-on direction as heat-induced degradation of the perovskite proceeds.

**Figure 3:**
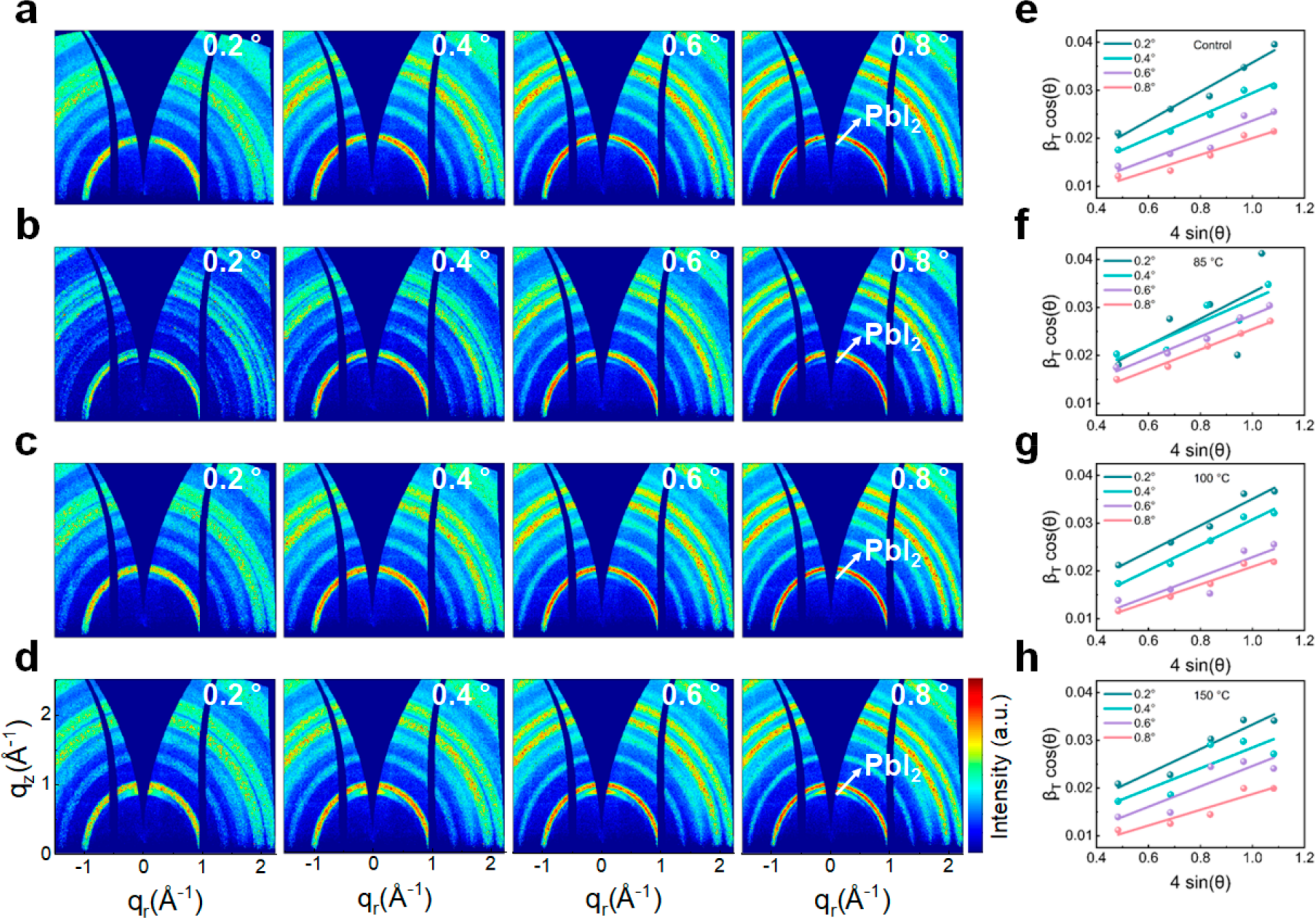
2D GIWAXS data of CsMAFA perovskite films measured at different probing depths (incident angles, α_i_) as a function of heating temperature for a fixed time of 60 min: (a) control, (b) 85 °C, (c) 100 °C, and (d) 150 °C. Calculation of the microstrain distribution in CsMAFA perovskite films measured at different probing depths (incident angles, α_i_) as a function of heating temperature for a fixed time of 60 min: (e) control, (f) 85 °C, (g) 100 °C, and (h) 150 °C.

In addition to probing the temporal evolution of the naked films at fixed temperature, we investigate the degradation process of the naked CsMAFA films as a function of temperature (control, 85 °C, 100 °C, and 150 °C) for a constant time of 60 min. Four representative incident angles, α_i_, of 0.2°, 0.4°, 0.6°, and 0.8° are used to gain insight into the crystal orientations and microstrain at different depths within the naked perovskite films. The in situ GIWAXS data and corresponding depth-dependent pseudo-XRD line profiles are shown in Figure 3a–d and Figure S8a–d, respectively. All films show similar Bragg rings in the 2D GIWAXS data and pseudo-XRD profiles, indicating that the films exhibit the same cubic phase structure at each of the different depths probed. However, we find that the intensity of PbI_2_ strongly depends on the heating temperature and depth within the films. With a fixed temperature, a well-defined Bragg ring belonging to PbI_2_ appears at 0.90 Å^–1^ in the 2D GIWAXS data as the probing depth increases, indicating that more diffraction signal is collected from the PbI_2_ located deeper inside the perovskite films. The PbI_2_ peaks are also observed in the pseudo-XRD profiles (Figure S8). With a fixed incident angle, α_i_, but increasing temperature, the intensity of the PbI_2_ Bragg peak strengthens and, accordingly, the Bragg ring becomes more pronounced.

At each fixed depth, the diffraction data are fitted with a Gaussian function to estimate the microstrain. The fit lines and calculated parameters are shown in Figure 3e–h and Table S4. With increasing probed depth, the fit lines show decreasing slopes for all heating temperatures. In particular, the extracted slopes decrease from 0.030 ± 0.003 to 0.017 ± 0.003 for the control sample, from 0.028 ± 0.019 to 0.021 ± 0.001 for 85 °C, from 0.027 ± 0.003 to 0.019 ± 0.002 for 100 °C, and from 0.026 ± 0.004 to 0.016 ± 0.003 for 150 °C heating for 60 min, implying that the microstrain in the naked CsMAFA films gradually decreases with increasing temperature. The variation in microstrain represents a compositional heterogeneity between the surface and the bulk. Moreover, the upper surface of the film is subjected to less microstrain at higher heating temperatures. For the heating temperatures of 85 °C, 100 °C, and 150 °C, the calculated slopes are 0.028 ± 0.019, 0.027 ± 0.003, and 0.026 ± 0.004 at α_i_ = 0.2° and 0.021 ± 0.001, 0.019 ± 0.002, and 0.016 ± 0.003 at α_i_ = 0.8°, respectively. These findings indicate that the microstain is inhomogeneous in naked CsMAFA perovskite films and follows a gradient distribution along the vertical direction within the films, wherein the top surface of the films suffer from the largest microstrain and the microstrain decreases from the top surface to the inner regions of the films. This finding is in good agreement with previous studies that identified the gradient distribution of the in-plane strain component perpendicular to the substrate surface in (FAPbI_3_)_0.85_(MAPbBr_3_)_0.15_ perovskite film.^10,27^

We further analyze the crystal stacking mode at different heating temperatures. The azimuthal tube cut data and corresponding Lorentz-corrected orientation distributions, including fits, are shown in Figures S9 and S10, respectively. For all of the studied temperatures, the intensity of the scattering signal at around χ ≈ ±60° becomes stronger and sharper with increasing probe depth. Thus, more intense signal is detected from crystallites located at deeper regions within the films and from those oriented in an edge-on direction. Despite this clear spatial distribution within the films, no significant correlation is observed between the heating temperature and the crystalline stacking pattern. However, we observe a gradual red-shift in the normalized PL spectra with increasing heating temperature (Figure S11a,b). The PL peak shifts from 766.50 ± 0.03 nm to 770.19 ± 0.02 nm (Table S5), indicating a gradually decreasing bandgap. This trend could be related to heat-induced structural changes in the mixed perovskite films, similar to those discussed above for the 150 °C time series.^23^

## Morphology and Crystal Structure Variation of CsMAFA Films under UV and 1 Sun Illumination

**Figure 4:**
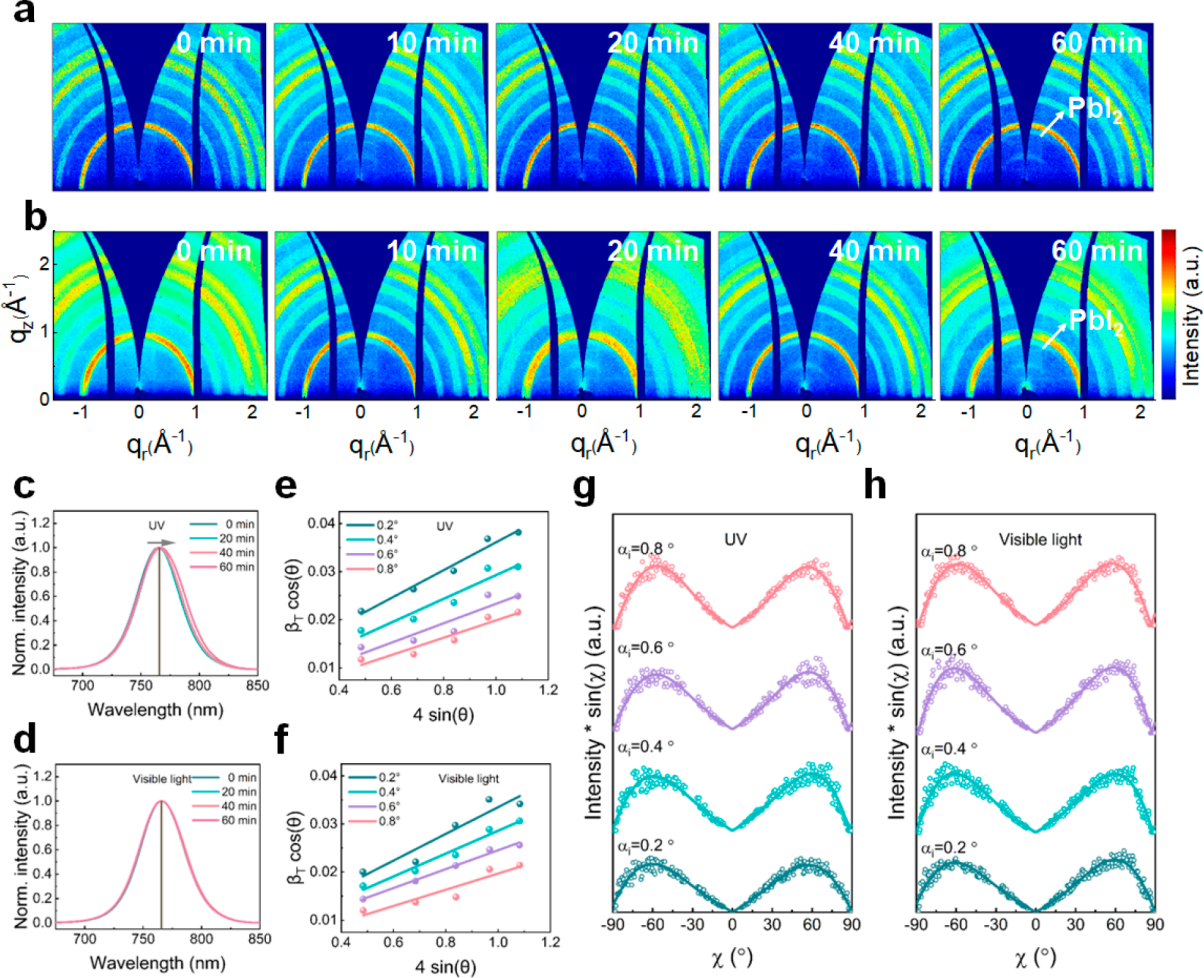
2D GIWAXS data measured at incidence angle α_i_ = 0.4° following sustained illumination with (a) UV and (b) visible (1 Sun) light as a function of time. PL spectra obtained as a function of time after sustained illumination with (c) UV and (d) visible (1 Sun) light. Calculation of microstrain distribution of CsMAFA films at different probing depths after exposure to (e) UV and (f) visible (1 Sun) light for 60 min. Pole figure representations of CsMAFA films at different probing depths, obtained after exposure to (g) UV and (h) visible (1 Sun) light for 60 min.

UV and visible light soaking also affect the operational stability of PSCs, playing important roles in degradation by unfavorably modifying charge carrier dynamics, promoting ion migration, and generating defects. Figures S12 and S13 show SEM images of naked CsMAFA perovskite films as a function of exposure time to UV and visible (1 Sun) light illumination, respectively. Surprisingly, compared with the pristine film, the UV- and visible light-exposed films exhibit denser and smoother morphologies, suggesting that these stimuli might reduce disorder in the films. To further investigate light-induced changes, we analyze 2D GIWAXS data collected after exposure to the same conditions (Figure 4a,b). These data reveal the emergence of a weak PbI_2_ signal in the UV- and visible light-treated films, which is further confirmed by the radial cake-cut data (Figure S14). Interestingly, a new Bragg ring is observed at *q* = 0.51 Å^–1^ with increasing illumination time. This new diffraction signal can be assigned to the solvent-coordinated perovskite intermediate phase (MAFA)Pb_3_I_8_·DMF, which is associated with the presence of residual solvent that is not effectively removed during the annealing step of the synthesis process. Thus, a reverse reaction of the intermediate solvent-coordinated complex conversion to the perovskite phase occurs under UV and visible light soaking. Such a diffraction signal is usually detected in unannealed mixed perovskite films.^21^ Thus, its observation here suggests the possibility of reversible reaction that might be leveraged for light-induced postsynthetic modifications, or even healing, of CsMAFA thin films.

According to the azimuthal tube-cut data along the (110)/(002) plane and the Lorentz-corrected pole figures containing Gaussian fit lines (Figures S15 and S16), all films show a preferential edge-on orientation at χ ≈ ±60°. The similarities of the fitted line profiles illustrate that UV- and visible light-soaking do not significantly affect the crystalline arrangement, which is consistent with observations of the heat-treated samples discussed above. Therefore, we conclude that the extrinsic environmental factors investigated here (heat, UV, and visible light) do not strongly affect the crystal orientations within annealed perovskite thin films. However, we note that other strategies, such as modification of the perovskite precursor composition or the film fabrication procedure, can be utilized to intentionally modulate crystal growth.^7^

Though UV illumination does not result in significant long-range structural reorganization, the PL spectra of naked CsMAFA perovskite films following UV exposure (Figure 4c) do indicate internal changes to the films. In particular, we observe a slight yet systematic red-shift of the PL emission with increasing UV illumination time, with the peak moving from 765.54 ± 0.04 nm to 767.41 ± 0.03 nm. The PL fit data and fit parameters are shown in Figure S17 and Table S6, respectively. Similar to the case of heating, this redshift can originate from the formation of the FA-rich perovskite phase accompanied by the loss of MA during UV soaking or from halide ion redistribution.^22,23^ However, this phenomenon is not observed in the PL spectra obtained after visible light soaking, with the PL emission maintaining around 766 nm (Figure 4d, Figure S18, and Table S7). This finding indicates that heat and UV can affect the ion redistribution with the perovskite films compared to visible light illumination at the same exposure time.

We further investigate the film structure at different detection depths under UV and visible light soaking. The corresponding 2D GIWAXS data measured at incidence angles of 0.2°, 0.4°, 0.6°, and 0.8°, as well as the extracted pseudo-XRD profiles, are shown in Figures S19 and S20, respectively. Similar to the case of heating, we observe a progressive strengthening of the PbI_2_ signal with increasing probing depth, suggesting that heat-, UV-, and visible light-degradation occur throughout the perovskite layer, rather than being limited to the top surface in direct contact with the environment. By performing linear fits of β_T_ cos θ vs 4sin θ (Figure 4e,f, corresponding fit parameters are listed in Table S4), we estimate the microstrain at incident angles α_i_ of 0.2°, 0.4°, 0.6°, and 0.8° to be 0.029 ± 0.003, 0.025 ± 0.004, 0.020 ± 0.005, and 0.018 ± 0.003 for UV-soaked films and 0.028 ± 0.005, 0.024 ± 0.002, 0.020 ± 0.001, and 0.017 ± 0.004 for visible light-soaked films, respectively. As observed under heating, the microstrain in the top region of the perovskite films is significantly higher than that inside the film. This finding further confirms the conclusion that the microstrain is gradient distributed along the vertical direction of the films and decreases from the upper surface to the inner regions of the perovskite films. The comparison of microstrain under heat, UV, and visible light conditions in terms of corresponding pseudo-XRD profiles and β_T_ cos θ vs 4sin θ data is summarized in Figures S21 and S22. At an incident angle of α_i_ = 0.2°, the estimated microstrains are 0.030 ± 0.003, 0.026 ± 0.004, 0.029 ± 0.003, and 0.028 ± 0.005 for control, heat, UV, and visible light, respectively. At an incident angle of α_i_ = 0.8°, the estimated microstrains are 0.017 ± 0.003, 0.016 ± 0.003, 0.018 ± 0.003, and 0.017 ± 0.004 for control, heat, UV, and visible light, respectively. Thus, we find that heat treatment appears to influence the microstrain at the surface but has a minimal impact on the microstrain inside the films. In contrast, UV and visible light treatments exhibit a slight effect on the film microstrain.^20^

## Effect of Heat, UV, and Visible Light on the Film Surface Topography and Surface Potential Distribution

**Figure 5:**
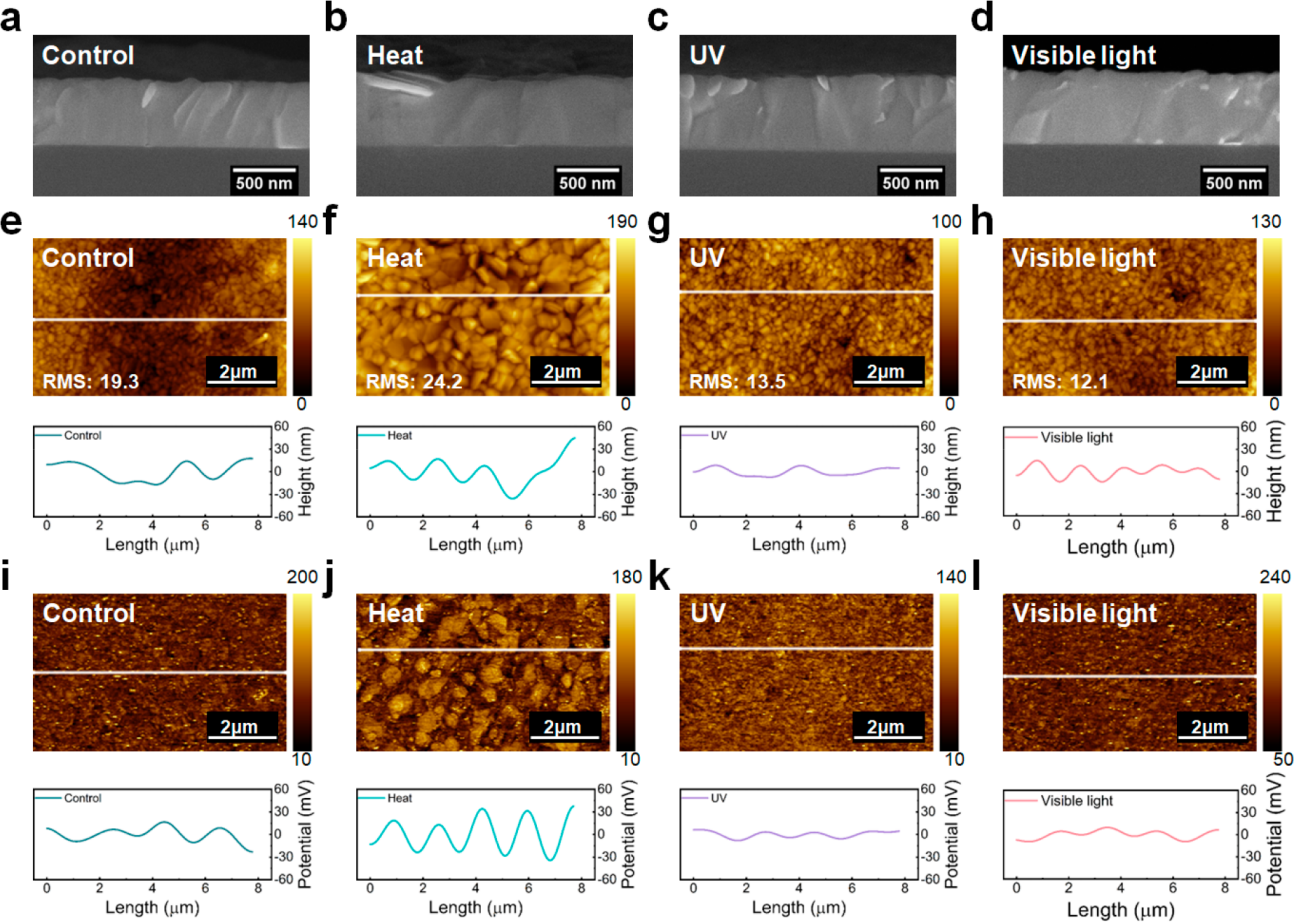
Nanoscale characteristics of CsMAFA perovskite films after exposure to different conditions for 60 min. Cross-sectional SEM images with a scale bar of 500 nm: (a) control, (b) heat at 150 °C, (c) UV, and (d) visible (1 Sun) light. AFM topography images with a scale bar of 2 μm and corresponding line profiles: (e) control, (f) heat at 150 °C, (g) UV, and (h) visible (1 Sun) light. KPFM surface potential images with a scale bar of 2 μm and corresponding line profiles: (i) control, (j) heat at 150 °C, (k) UV, and (l) visible (1 Sun) light.

Figure 5a–d presents the cross-sectional SEM images of CsMAFA perovskite films exposed to different conditions for 60 min. As discussed above, after heat-treatment, the well-defined grains merge into larger grains. In contrast, no obvious variation is found for the case of UV- and visible light-treated films. These results are consistent with top-view SEM results (Figures 2a, S2, S12, and S13). To gain better insight into nanoscale changes to the films, we conduct atomic force microscopy (AFM) and Kelvin probe force microscopy (KPFM) to examine the surface topography and surface potential distributions, respectively, of naked CsMAFA perovskite films under external stressors. Figure 5e–h shows the AFM images and corresponding line profiles. After annealing at 150 °C for 60 min, the film becomes rougher, with an increase in the root-mean-square (RMS) roughness from 19.3 to 24.2 nm. In contrast, the UV- and visible light-treated films exhibit significantly smoother morphologies, with the RMS roughness decreasing to 13.5 and 12.1 nm, respectively. This difference is also reflected in the line scans of the topography images (Figure 5e–h, lower panels), where larger height differences are observed in heat-treated films.

KPFM provides a reliable measurement of local surface potentials stemming from contact potential differences between the tip and the sample surface associated with their relative work functions.^28^ The surface potential distribution can be affected by various factors, including the presence of surface defects, composition inhomogeneities, and modified adsorbates or surface terminations.^13^ The surface potential maps shown in Figure 5i–l indicate that heat-treatment causes strong surface potential fluctuations (Figure 5j) compared to the control (Figure 5i), as well as the UV (Figure 5k) and visible light (Figure 5l) exposed samples. In particular, as seen from the line scan of surface potential variations from a typical perovskite region (Figure 5i–l, lower panels), the potential fluctuation varies over an approximate peak-to-peak range of 18, 29, 4, and 8 mV for control, heat-, UV-, and visible light-treatment samples over a length scale of about 8 μm, respectively. The extracted RMS values is 21.30, 27.24, 15.84, and 21.27 mV for control, heat-, UV-, and visible light-treatment samples over an area of 10 μm × 10 μm. The increase in the surface roughness and potential distribution for the heat-treated perovskite film can be attributed to thermal-degradation and defect generation.^13,29^ Exposure to elevated temperatures leads to grain coarsening and the degradation of perovskite into PbI_2_ within the films (Figures 2a,b, S2, and S3). This transformation results in strong fluctuations in the film flatness and the local surface potential. In contrast, we speculate that the short UV and visible light immersion provides a self-optimization effect, wherein small crystal coalescence occurs through Ostwald ripening under continuous illumination. This process promotes mass transfer between grains, yielding smoother perovskite thin films with reduced surface roughness and more uniform surface potential distributions (Figures S12 and S13, SEM).^30^ Similarly, the crystal coalescence also occurs in thermal conditions. However, findings on the film surface topography and surface potential distribution of naked perovskite films may not fully capture the performance and behavior of real devices. The introduction of charge transport layers, electrodes, and encapsulants may change this conclusion.^7^

**Figure 6:**
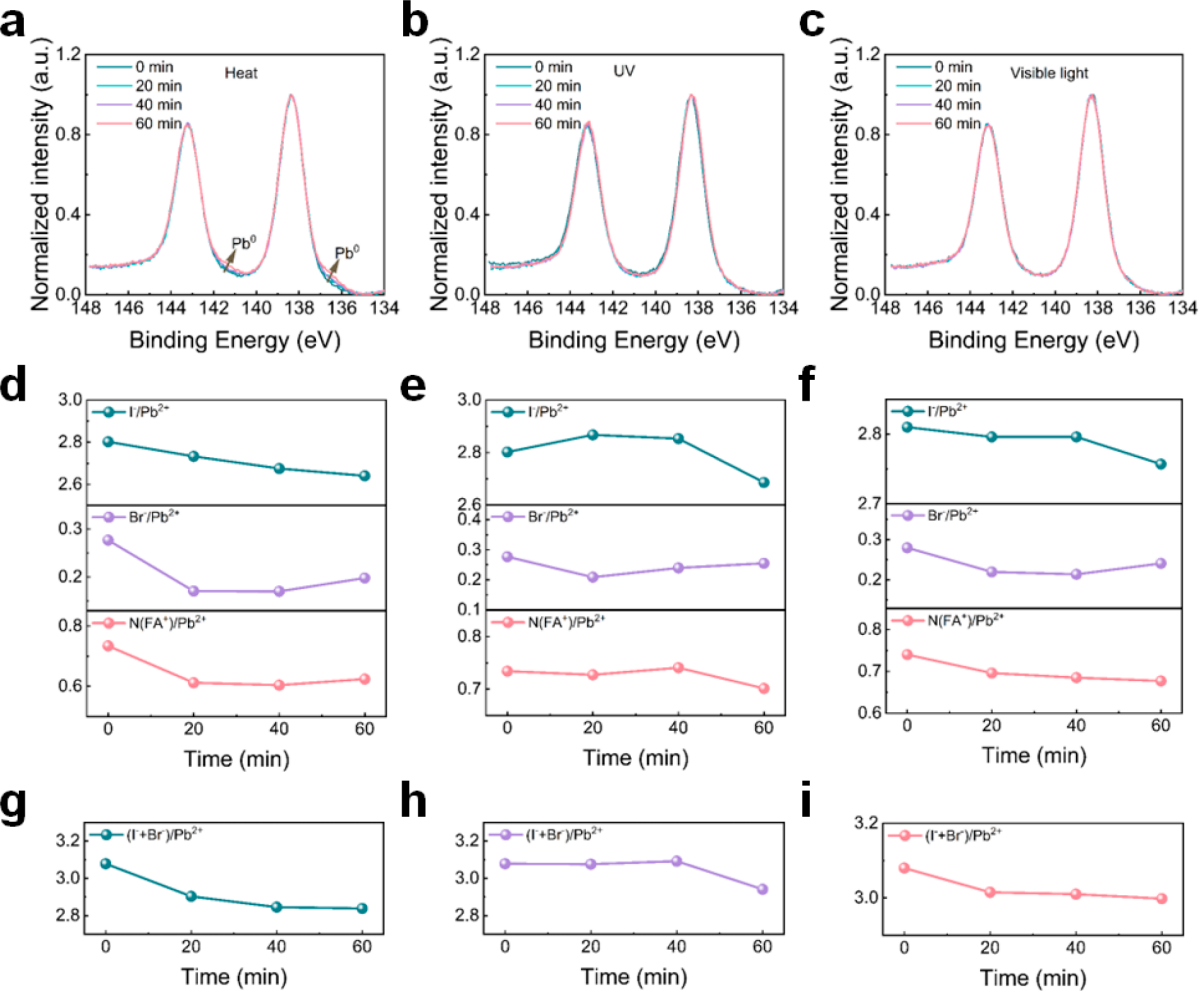
Time-dependent Pb 4f core level XPS spectra of CsMAFA perovskite films exposed to different conditions: (a) heat, (b) UV, and (c) visible light (1 Sun). I^–^/Pb^2+^, Br^–^/Pb^2+^, and N(FA^+^)/Pb^2+^ ratios as a function of exposure time under different conditions: (d) heat, (e) UV, and (f) visible (1 Sun) light. Changes of the (I^–^ + Br^–^)/Pb^2+^ ratios as a function of exposure time under conditions of (g) heat, (h) UV, and (i) visible (1 Sun) light.

With XPS, we next determine the chemical composition at the surfaces of the perovskite films under different external stressors. Figure S23 shows the XPS spectra from the I 3*d*, Pb 4*f*, Br 3*d*, and N 1*s* core level regions under heat, UV, and visible light conditions for different times, with the associated peak assignments provided in Tables S8, S10, and S12. For the I 3*d* XPS spectra, the spin–orbit split peaks are located at 619.2 eV (I 3*d*_5/2_) and 630.7 eV (I 3*d*_3/2_), which can be assigned to I^–^. In the Pb 4*f* region, the spin–orbit slit peaks are located at 138.3 eV (Pb 4*f*_7/2_) and 143.3 eV (Pb 4*f*_5/2_), which are associated with Pb^2+^.^31^ The Br 3*d*_5/2_ and Br 3*d*_3/2_ peaks are located at 68.4 and 71.5 eV, respectively, which correspond to Br^–^. The peak located at 400.4 eV in N 1*s* spectra is typically attributed to the formamidinium (FA^+^) cation.^31^ A signal for methylammonium (MA^+^) cation would be expected at around 402.5 eV; however, we are unable to reliably quantify this species due to the low concentrations. No changes in the core level binding energies are observed for any of the exposure conditions. Additionally, small shoulders are observed in the Pb 4*f* region at lower binding energies of 136.7 and 141.6 eV for the as-grown films, which indicate the formation of Pb^0^ most likely formed during sample fabrication and degradation (Figure S24).^32^ For the sample exposed to heat, the Pb^0^ peak intensity increases with heating time (the proportion of component increases from <1% to 2%, Table S8), indicating that metallic Pb^0^ is formed during heating (Figure 6a). The formed Pb^0^ suggests that a small portion of Pb shifts its chemical state from Pb^2+^ (138.3 eV) to Pb^0^ (136.7 eV) as heat-induced degradation of the perovskite proceeds.^32^ However, no obvious increase in the signal from metallic Pb^0^ is detected in the samples exposed to UV and visible light illumination for 60 min (Figure 6b,c). This finding corresponds to the phenomenon observed in the GIWAXS data and SEM images for samples exposed to heat. That is, heat induces severe degradation of the perovskite components and leads to the generation of Pb-based inorganic components (PbI_2_ and Pb^0^), as seen in GIWAXS and XPS data compared with UV and visible light. We note that, since a nonmonochromatic X-ray source is used, the possible formation of PbI_2_ could not be quantified due to spectral overlap.

Alongside the subtle changes to the perovskite surface species described above, significant changes to the elemental ratios are observed as a function of time under different treatments. Figure 6d–f shows the calculated I^–^/Pb^2+^, Br^–^/Pb^2+^, and N(FA^+)^/Pb^2+^ ratios for specific species as a function of exposure time. In particular, the calculated I^–^/Pb^2+^ ratios decrease with increasing heating time at 150 °C (Figure 6d, Tables S8 and S9), yielding values of 2.8, 2.7, 2.7, and 2.6 for 0, 20, 40, and 60 min heating. Likewise, the calculated Br^–^/Pb^2+^ ratios are given by 0.3, 0.2, 0.2, and 0.2 for the same conditions, respectively. The reduction in I^–^/Pb^2+^ and Br^–^/Pb^2+^ ratios is associated with the loss of univalent cations (MA^+^ and FA^+^), which is discussed below. The loss of organic cations is confirmed by the evolution of N(FA^+^)/Pb^2+^ ratio, plotted in Figure 6d, which decreases from 0.7 to 0.6 with increasing heating time.

While a continuous decrease of the halide and organic components is observed during heating, a different behavior is observed for the case of UV exposure. In particular, when the films are exposed to UV illumination (Figure 6e, Tables S10 and S11), the I^–^/Pb^2+^ ratio first increases from 2.8 to 2.9 and then decreases to 2.7. This phenomenon is less pronounced for the case of visible light treatment, where the I^–^/Pb^2+^ ratio remains approximately constant at around 2.8 (Figure 6f and Tables S12 and S13). While this behavior was initially unexpected, we hypothesize that the initial rise in the I^–^/Pb^2+^ ratio could be caused by I^–^ ion migration toward the surface, assuming a constant Pb concentration at the surface (see discussion below). In particular, due to their low formation and migration energies, intrinsic ionic defects including vacancies and interstitials are prone to form and migrate under the external influence of heat, light, and electric fields.^33,34^ Compared with MA^+^ (*E*_a_ = 0.84 eV) and Pb^2+^ (*E*_a_ = 2.31 eV^35^), the I^–^ ions possess the lowest activation energy of 0.58 eV and shortest migration distance of 4.46 Å. Therefore, they are regarded as the fastest diffusing species and correspondingly are more prone to migrate, whereas the Pb^2+^ ions are unlikely to migrate.^35,36^ UV and visible light irradiation decrease the activation energies for ion migration and drive the I^–^ ions from the interior toward the interface, resulting in an increased I^–^/Pb^2+^ ratio.^36^ As the illumination time increases, the UV- and visible light-induced degradation lead to the generation of PbI_2_ (confirmed by GIWAXS data) and MAI, with the latter decomposing into HI and escaping from the film surface together with the release of MA.^31^ Accordingly, a decreased I/Pb ratio is detected with increasing illumination time following the initial halide redistribution. As more iodine escapes from the film surface, Br^–^ ions can diffuse to the film surface to balance the charge concentration and occupy the vacancies left by the escaping I^–^ ions.^37^ Therefore, we observe an increased Br^–^/Pb^2+^ ratio after 40 min of exposure to heat, UV, and visible light conditions. This hypothesis is consistent with the changes of Br^–^/I^–^ ratios, which first decrease and then increase as a function of time during UV and visible light exposure conditions (Figure S25 and Tables S11 and S13). We believe that the initial decrease could originate from the migration of I^–^ ions to the surface, and the later increase may be the result of the loss of I species and the migration of Br^–^ ions to surface. From the curves of overall halide/Pb ratio as a function of exposure time, we find a significant loss of halogen elements during heating, with a decrease of the total halide-to-lead ratio from 3.1 to 2.8 (Figure 6g and Table S9). In contrast, the (I^–^ + Br^–^)/Pb ratio remains more stable under UV and visible light illumination, decreasing from 3.1 to 2.9 under UV and from 3.1 to 3.0 under visible light illumination (Figure 6h,i and Tables S11 and S13). This change is in agreement with the findings we discussed in the context of Figure 6d–f, wherein the observed halide losses induced by thermal-degradation are greater than for UV and visible light-degradation.

Overall, the variation in I^–^/Pb^2+^, Br^–^/Pb^2+^, and N(FA^+^)/Pb^2+^ ratios reflects the dynamics of two simultaneous process involving perovskite phase degradation and ion migration. Under exposure to heat, the I^–^ ion loss is more rapid than the accumulation of I^–^ via ion migration. Thus, a continuously decreasing I^–^/Pb^2+^ ratio at the surfaces of the perovskite films is observed (Figure 6d). However, under UV and visible light illumination, the increased and stabilized I^–^/Pb^2+^ ratio in the first 40 min of exposure increase from 2.8 to 2.9 for UV and remain stable at around 2.8 for visible light (Tables S11 and S13). This finding most likely originates from photoinduced I^–^ ion migration that is initially more rapid than iodide species loss caused by degradation. Indeed, under UV illumination, the N(FA^+^)/Pb^2+^ ratios of approximately 0.7 exhibit no significant changes within the first 40 min of illumination, suggesting negligible chemical decomposition during this time (Figure 6e). However, after longer periods of exposure to UV light, the slightly decreased N^–^/Pb^2+^ ratio indicates the formation and escape of volatile organic components, resulting in degradation of the film surface. Since the rate of MA^+^ and FA^+^ migration within the perovskite film is comparatively slow, it cannot compensate for the losses caused by the decomposition process, which is especially pronounced under sustained heating. In addition, we find that in all cases the Br^–^/Pb^2+^ ratio decreases during the initial period of exposure and gradually increases thereafter. The increased Br^–^/Pb^2+^ ratio is attributed to imbalanced charge density caused by the I^–^ ion loss at the surface, which drives the diffusion of Br^–^ ions toward this region. The migration of I^–^ and Br^–^ ions, in turn, is associated with anion redistribution into I-rich and Br-rich regions, leading to slight red-shifts observed in PL spectra (Figure 2d and Figure 4c). In this model,^34^ it outlines the series of microscopic events that a halide experiences during its oxidation-mediated redistribution and provides new insights into the fundamental origins of halide oxidation and the thermodynamic and kinetic gradients that subsequently drive their redistribution. Ionic charge balance could be maintained by the halide ions drift or diffusion as reported recently.^34,38^ Through the above discussion, we find that heat (150 °C) has the largest impact on the decomposition of CsMAFA perovskite films and induces severe phase separation and ion migration.

In summary, we perform GIWAXS, complemented by XPS, AFM, and KPFM, to monitor the evolution of the crystal structure and the microstrain in naked triple-cation mixed halide perovskite CsMAFA films aged under heat, UV, and visible light stressors. We find that none of these factors affect the crystal orientations. However, all three conditions decrease the microstrain on the film surface, though minimal microstrain relaxation occurs deeper within the films and microstrain remains distributed over a gradient in the vertical direction. Heat-induced degradation leads to grain coarsening, which is accompanied by the generation of PbI_2_, film roughening, and the emergence of strong surface potential fluctuations arising from severe component degradation and phase separation. In contrast, our measurements reveal that short-term exposure to UV and visible light improves film homogeneity, reducing the film surface roughness, and inducing a more uniform potential distribution. In contrast, prolonged UV or visible light exposure drives ion migration and eventually triggers component degradation. Thus, these results shed light on dynamic processes induced by the major stressors on light-absorbing perovskites in solar cells, providing insight into initial performance enhancements under illumination as well as long-term degradation mechanisms.

## Supporting Information Available

Materials and preparation methods of perovskite films and solar cells; characterization methods of perovskite films and devices, including SEM, GIWAXS, PL, KPFM, and XPS; additional characterization data and parameters, including *J*–*V* data, SEM, pseudo-XRD, azimuthal tube-cut data, PL spectra, microstrain, and peak information on XPS (PDF)
