# Supplementary material for: Microstrain and Crystal Orientation Variation within Naked Triple-Cation Mixed Halide Perovskites under Heat, UV, and Visible Light Exposure
Source: ACS Energy Lett. 2024 Jan 8;9(2):388–99. doi: 10.1021/acsenergylett.3c02617 (PMC10863397; doi:10.1021/acsenergylett.3c02617)
Supplement: Supplementary file 1 — nz3c02617_si_001.pdf [file nz3c02617_si_001.pdf]

Supporting Information

Microstrain and crystal orientation variation within naked triple-cation mixed halide perovskites under heat, UV, and visible light exposure

Yuqin Zou[a], Johanna Eichhorn[b], Jiyun Zhang[c], Fabian A. C. Apfelbeck[a], Shanshan Yin[a], Lukas Wolz[b], Chun-Chao Chen[d], Ian D. Sharp[b], and Peter Müller-Buschbaum[a, e*]*

[a]Technical University of Munich, TUM School of Natural Sciences, Department of Physics, Chair for Functional Materials, James-Franck-Str. 1, 85748 Garching, Germany

[b]Walter Schottky Institute, Technische Universität München, 85748 Garching, Germany

Physics Department, TUM School of Natural Sciences, Technische Universität München, 85748 Garching, Germany

[c]Forschungszentrum Jülich GmbH, Helmholtz-Institute Erlangen-Nürnberg (HI ERN), Immerwahrstraße 2, 91058 Erlangen, Germany

[d]School of Materials Science and Engineering, Shanghai Jiao Tong University, Shanghai 200240, P. R. China

[e]Technical University of Munich, Heinz Maier-Leibnitz-Zentrum (MLZ), Lichtenbergstr. 1, 85748 Garching, Germany

Experimental Methods

Materials

Indium-doped tin oxide (ITO)-coated glass substrates with size of 25×25 mm^2 were purchased from Lumtec. Hellmanex solution was purchased from Sigma-Aldrich. Tin oxide (SnO_2, 15% in H_2O colloidal dispersion) precursor was purchased from Alfa Aesar. Formamidinium iodide (FAI), lead iodide (PbI_2), and lead bromide ($PbBr_2$) were purchased from TCI Chemicals. Methylammonium bromide (MABr), cesium iodide (CsI), *N, N*-dimethylformamide (DMF), dimethyl sulfoxide (DMSO), chlorobenzene (CB), 2-propanol (IPA), lithium bis(trifluoromethylsulphonyl)imide solution (LiTFSI) and 4-tert-butylpyridine (TBP) were purchased from Sigma Aldrich. The LED UV lamp was purchased from PEREL and the UV wavelength was 395-400 nm.

Films and solar cell device fabrication

The ITO-coated substrates were cleaned by ultrasonic cleaner for 25 min, sequentially in diluted Hellmanex solution ($V_{Hellmanex}$:$V_{DI\text{-}water}$, 2:98), deionized water, acetone, and isopropanol. Before use, the ITO-coated substrates were dried using a nitrogen gun and treated with UV-ozone for 10 min. The SnO_2 precursor solution (V_{SnO2}:V_{H2O}, 1:4) was stirred for 2 h, filtered, and spin-coated onto the ITO substrates at 4000 rpm for 30 s, followed by annealing in ambient air at 150 °C for 30 min. After cooling down, the substrates were UV-ozone treated again for 10 min and then transferred into a nitrogen-filled glovebox. For the CsMAFA perovskite precursor solution, FAI (1.19 M), PbI_2 (1.30 M), CsI (0.07 M), MABr (0.20 M), and $PbBr_2$ (0.20 M) were dissolved in 1 mL mixed solvent of DMF and DMSO (V_{DMSO}: V_{DMF}, 1: 4) and stirred for 2 h. The solution

was then spin-coated on ITO/ SnO_2 substrates at 1000 rpm for 5 s and 5000 rpm for 20 s. During the second spin-coating process, 130 μL chlorobenzene was quickly dropped into the center of the substrates at 10 s before the end of the program. Subsequently, the perovskite films were annealed at 100 °C for 60 min. A spiro-OMeTAD-based solution was then prepared by mixing 72.3 mg of spiro-OMeTAD in 1 ml chlorobenzene along with 30 μl of TBP and 35 μl of a Li-TFSI solution (260 mg ml^{-1} in acetonitrile), and was then dynamically spin-coated onto the surface of the perovskite films at 3000 rpm for 30 s. Finally, a gold electrode was thermally evaporated with a thickness of 80 nm at a low vacuum pressure of 2×10^{-6} bar. The evaporation speed was 0.2 Å s^{-1} for the first 10 nm and 0.8-0.9 Å s^{-1} up to the final thickness of 80 nm. Following fabrication, the devices were kept in a desiccator and were measured regularly until the efficiency reached stable values.

Perovskite film characterization

Scanning electron microscopy (SEM): The surface morphology was probed by a field-emission SEM (Zeiss NVision 40) with an acceleration voltage of 5 kV and a working distance of 3.2 mm.

Atomic force microscopy (AFM): The film roughness and contact potential difference were obtained using an AFM instrument (Core AFM from nanosurf. Tip: radius = 10 nm, cantilever: f_{res} = 190 kHz) in tapping mode using conical tips. For the AFM measurement, trapping mode AFM probes with long cantilevers and aluminum reflective coating (Tap 190Al-G) were used. For the KPFM measurement, force modulation mode was applied using AFM probes with platinum coatings

(Electrimulti75-G). The measurements were performed under ambient conditions, and the excitation amplitude was 2 V.

Steady-state photoluminescence (PL): The perovskite films deposited on Si were excited with a wavelength of 450 nm and the measured emission wavelength range was set to 400 to 900 nm. The scanning speed was set to 200 nm/min.

Grazing incidence wide-angle X-ray scattering (GIWAXS): The GIWAXS measurements were conducted in-house at TU Munich under vacuum using a Ganesha SAXSLAB instrument. The X-ray wavelength was 1.54 Å, the sample-to-detector distance was 95.67 mm, the incident angle α_i was set to 0.2°, 0.4°, 0.6°, and 0.8° to obtain information at different depths within the samples. The scattering signals for all X-ray measurements were recorded with a Pilatus 300 K detector (Dectris Ltd) with a pixel size of 172 μm ×172 μm. In the GIWAXS measurements, a resolution of $\Delta q \approx 0.013$ Å^{-1} was achieved and the direct beam was blocked with the beamstop to protect the detector from oversaturation. The 2D detector image was a distorted version of the reciprocal crystal lattice, with corrections including solid-angle correction, efficiency correction, and polarization performed with the Matlab-based software GIXSGUI to reconstruct the raw 2D GIWAXS data into the reciprocal-space coordinate system. For analyzing the crystallinity and orientation of the samples, radial cake cuts for pseudo-XRD were completed with χ = [-90°, 90°] and q = [0, 3] Å^{-1} as cut limits. Azimuthal tube cuts for orientation analysis were completed with χ = [-90°, 90°] and suitable q range covering the whole diffraction ring width, which was also referring to the Bragg peaks in the pseudo-XRD profiles.

X-ray photoelectron spectroscopy (XPS): XPS was conducted to analyze the near-surface composition using a non-monochromatized Al Kα source (hν = 1486.6 eV) and a pass energy of 20 eV on a SPECS system. The spectra were fitted using Casa XPS analysis software with Shirley background. The binding energy was calibrated by shifting the C 1s to 284.8 eV.

Solar cell characterization

J-V measurement: The current density–voltage (*J–V*) data were collected using a Keithley 2611B source meter under the illumination of a solar simulator (class ABA) with a light intensity of 100 mW cm^{-2}. Before device measurements, the intensity of the solar simulator was measured using a reference solar cell (Fraunhofer ISE019-2015), and the recorded intensity was used to calculate the precise power conversion efficiency. Every device contained six gold top electrodes with a dimension of 2.5×10.0 mm^2 on a substrate with a size of 25×25 mm^2. The active area was defined by a metal aperture mask (0.1 or 0.1875 cm^2) to avoid the high photocurrent caused by edge effects. The *J-V* curves were measured with a scanning speed of 50 mV s^{-1} (integration time of 100 ms) from 1.25 V to -0.20 V and then back again (from -0.20 V to 1.25 V). The cells were measured several times to reach the highest PCE for each.

Supplementary Note 1. Crystallite size and microstrain calculation from GIWAXS line cuts data using Williamson-Hall (W-H) method[1]

The broadening (β_T) of the peaks is due to the combined effect of the crystallite size (β_D) and microstrain (β_ε) according to:

$$\beta_T = \beta_D + \beta_\varepsilon \qquad (1)$$

where β_T is the total broadening, β_D is broadening due to crystallite size, and β_ε is the broadening due to stain.

From the Scherer equation, β_D is expressed as:

$$\beta_D = \frac{K\lambda}{D\cos\theta} \qquad (2)$$

where β_D is the FWHM (broadening of the peak) in radians, $K = 0.89$ is the shape factor, $\lambda = 0.15406$ nm is the wavelength of X-rays, D is the crystallite size, and θ is the peak position in radians.

Similarly, the microstrain is given by:

$$\beta_\varepsilon = 4\varepsilon\tan\theta \qquad (3)$$

where β_ε is broadening due to strain, ε is the strain, and θ is the peak position in radians.

Combining equation (2) and (3) into (1), one obtains:

$$\beta_T = \frac{K\lambda}{D\cos\theta} + 4\varepsilon\tan\theta \qquad (4)$$

and thus:

$$\beta_T\cos\theta = \frac{K\lambda}{D} + 4\varepsilon\sin\theta \qquad (5)$$

in which ε is the gradient (slope) of the line, and $\frac{K\lambda}{D}$ is the y-intercept.

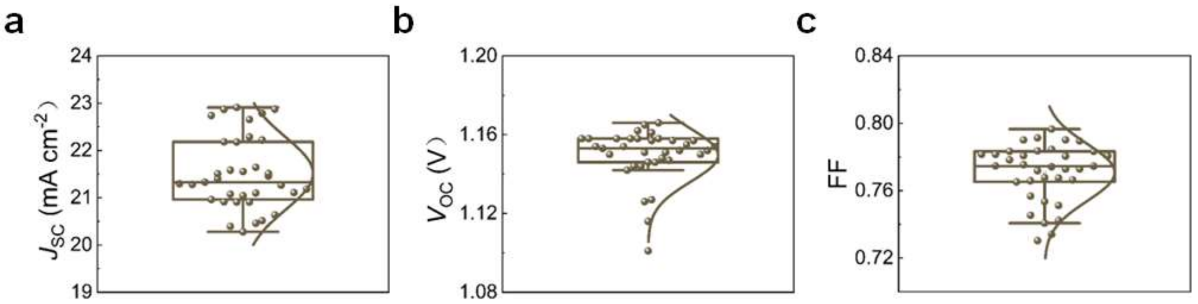

Figure S1. Statistical distribution of photovoltaic parameters determined from the *J–V* curves for 35 cells, (a) J_{SC}, (b) V_{OC}, (c) FF.

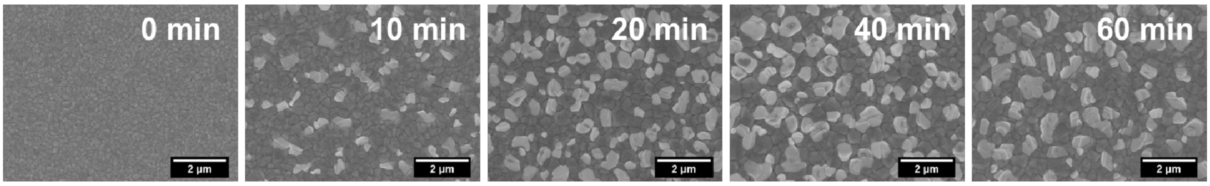

Figure S2. SEM images of CsMAFA perovskite films at different heating times of 0 min, 10 min, 20 min, 40 min, and 60 min, all with a heating temperature of 150 °C. The scale bar is 2 μm long for all images.

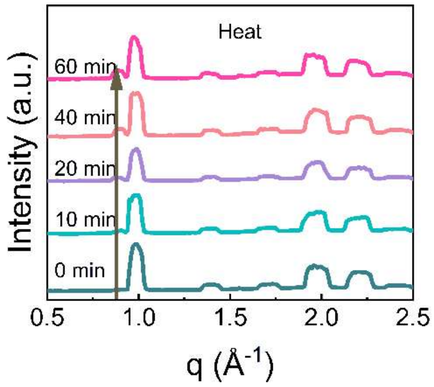

Figure S3. Pseudo-XRD data extracted from the 2D GIWAXS data by radial cake cuts measured at different heating times, with a heating temperature of 150 °C.

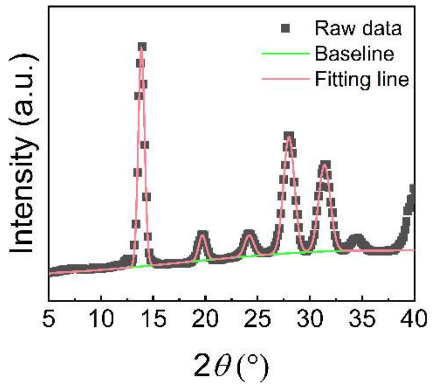

Figure S4. Pseudo-XRD data extracted from the 2D GIWAXS data by radial cake cuts (black squares) and the corresponding Gaussian fit (red line) for microstrain calculation.

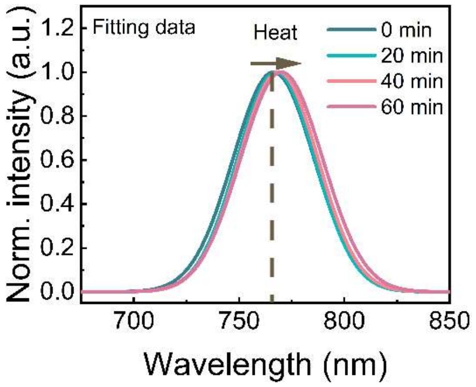

Figure S5. PL fitting data of CsMAFA films heated at 150 °C for different times.

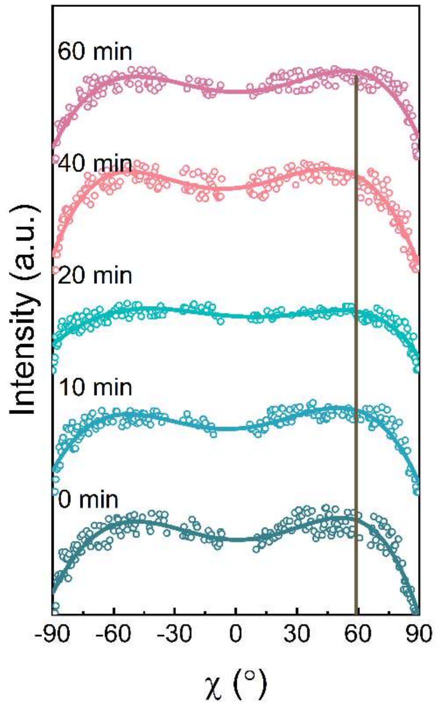

Figure S6. Azimuthal tube cuts data (circles) and the corresponding Gaussian fits (lines) extracted from the (002/110) Bragg peak at around q = 1.00 Å^{-1} of 2D GIWAXS data measured at different heating times. The incident angle α_i is 0.40° and the heating temperature is 150 °C.

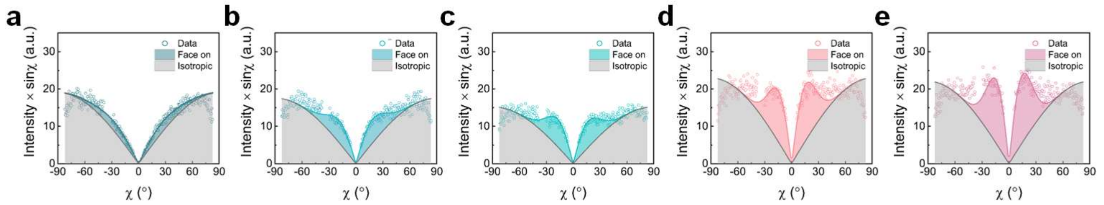

Figure S7. Pole figure representation of PbI_2 phase at incident angle α_i of 0.40°, with different color areas corresponding to the face-on and isotropic material quantity *MQ*, respectively. Data were collected as a function of heating time of (a) 0 min, (b) 10 min, (c) 20 min, (d) 40 min, and (e) 60 min, all at a constant temperature of 150 °C.

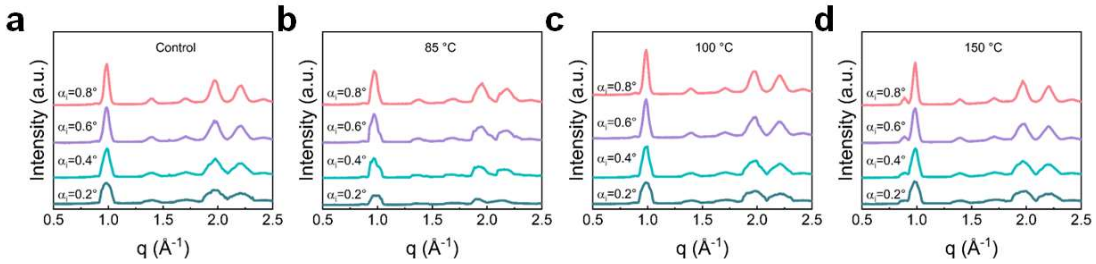

Figure S8. Pseudo-XRD data extracted from the 2D GIWAXS data by radial cake cuts measured at different incidence angles α_i and heating temperatures for 60 min: (a) control, (b) 85 °C, (c) 100 °C, and (d) 150 °C.

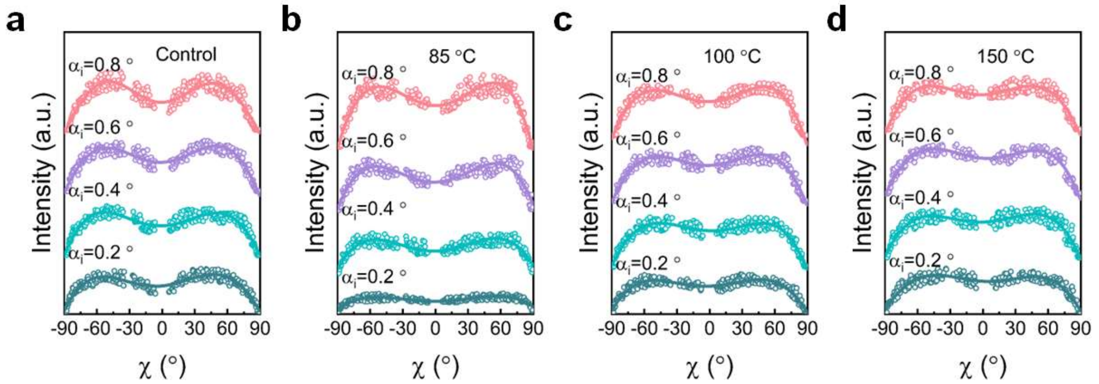

Figure S9. Azimuthal tube cut data (circles) and the corresponding Gaussian fits (lines) extracted from the (002/110) Bragg peak at around q = 1.00 Å^{-1} of the 2D GIWAXS data measured at different incident angles α_i and heating temperatures for 60 min: (a) control, (b) 85 °C, (c) 100 °C, and (d) 150 °C.

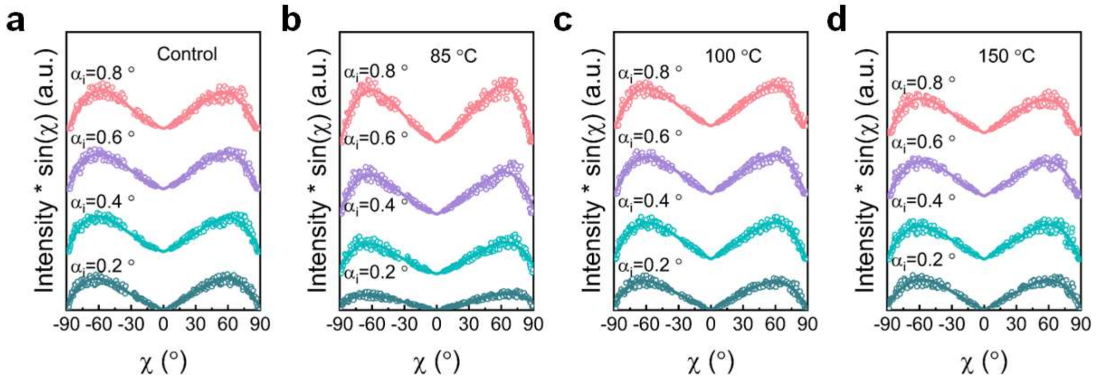

Figure S10. Pole figure representations of the corresponding perovskite films at different incident angles α_i and heating temperatures for 60 min: (a) control, (b) 85 °C, (c) 100 °C, (d) 150 °C.

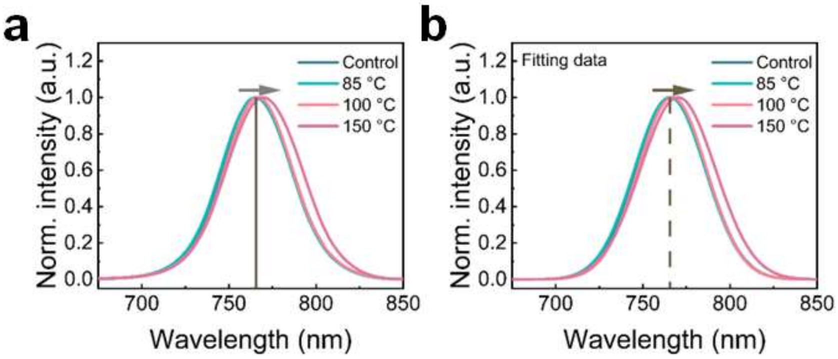

Figure S11. CsMAFA perovskite films heated for 60 min at different temperatures, (a) PL spectra, (b) PL fitting data.

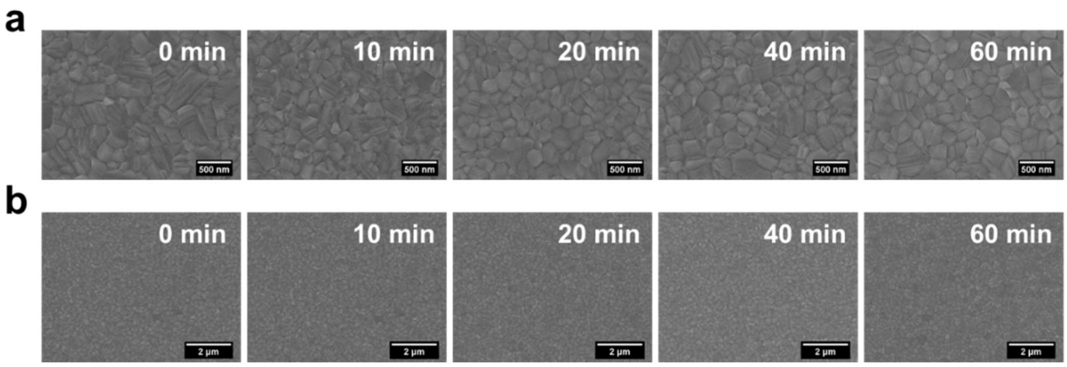

Figure S12. Top-view SEM images of CsMAFA perovskite films soaked under UV illumination for different times indicated in each panel, (a) with a scale bar of 500 nm, (b) with a scale bar of 2 μm.

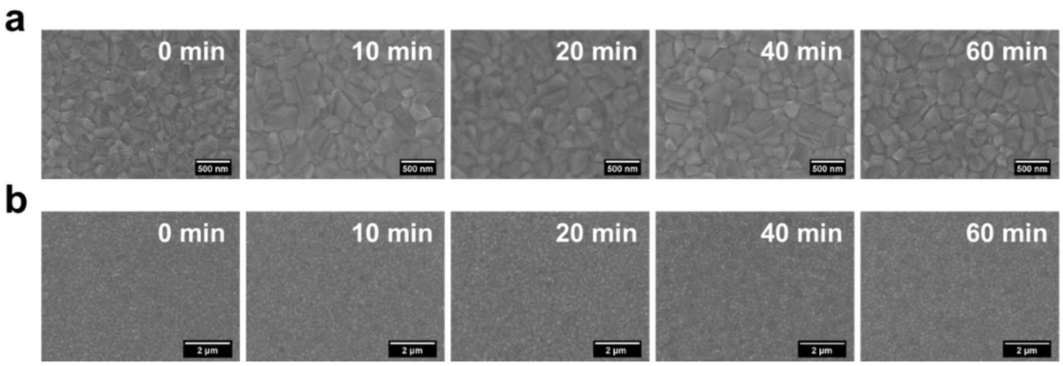

Figure S13. Top-view SEM images of CsMAFA perovskite films soaked under visible light (1 Sun illumination) for different times indicated in each panel, (a) with a scale bar of 500 nm, (b) with a scale bar of 2 μm.

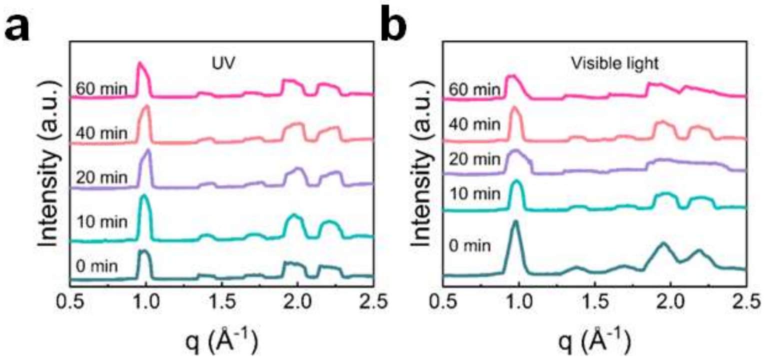

Figure S14. Pseudo-XRD extracted from the 2D GIWAXS data by radial cake cuts, (a) under UV exposure and (b) under visible light (1 Sun) exposure.

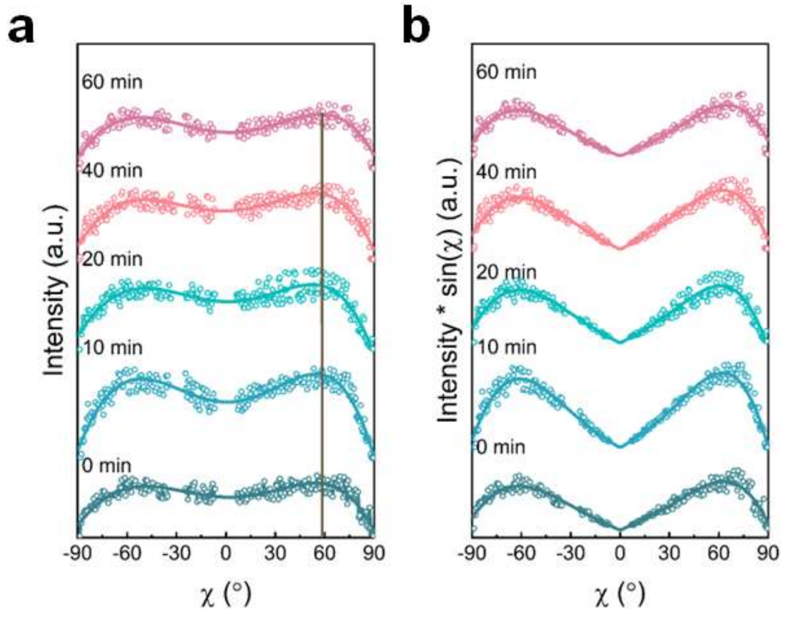

Figure S15. (a) Azimuthal tube cut data (circles) and the corresponding Gaussian fits (lines) extracted from the (002/110) Bragg peak at around q =1.00 Å^{-1} of the 2D GIWAXS data measured at different UV illumination times, and with constant incident angle α_i of 0.40°. (b) Pole figure representation of the corresponding perovskite films, with different colored areas corresponding to the edge-on and isotropic material quantity *MQ*, respectively.

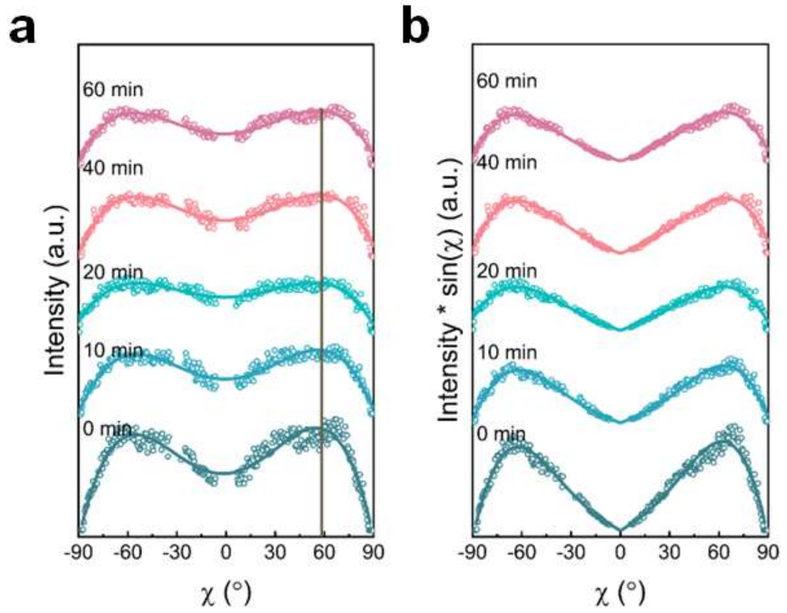

Figure S16. (a) Azimuthal tube cut data (circles) and the corresponding Gaussian fits (lines) extracted from the (002/110) Bragg peak at around q =1.00 $Å^{-1}$ of the 2D GIWAXS data measured at different visible light (1 Sun) illumination times, with constant incident angle α_i of 0.40°. (b) Pole figure representation of the corresponding perovskite films, with different colored areas corresponding to the edge-on and isotropic material quantity *MQ*, respectively.

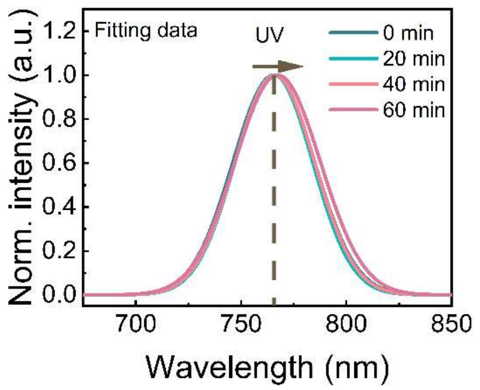

Figure S17. PL fitting data for CsMAFA films immersed under UV light for different times.

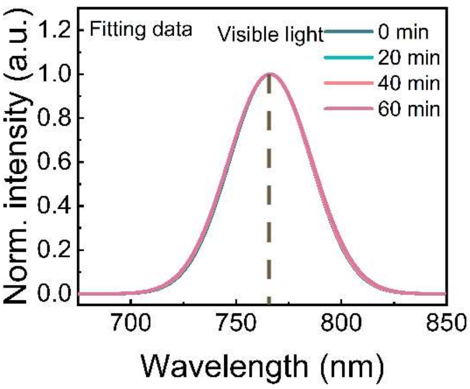

Figure S18. PL fitting data for CsMAFA films immersed under visible light for different times.

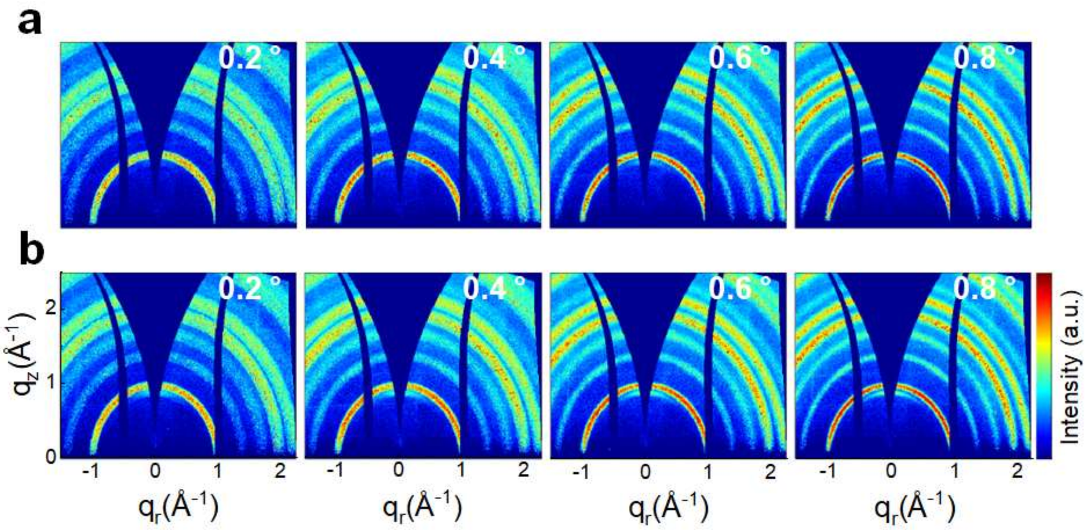

Figure S19. 2D GIWAXS data of CsMAFA perovskite films under different conditions for 60 min of exposure to (a) UV and (b) visible light (1 Sun) illumination. Measurements were performed at different incident angles, α_i, as indicated in each panel.

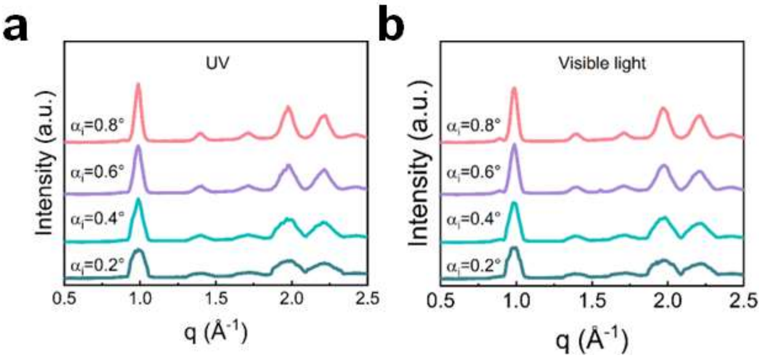

Figure S20. Pseudo-XRD data extracted from the 2D GIWAXS data by radial cake cuts measured at different incidence angles α_i under (a) UV and (b) visible light (1 Sun) illumination for 60 min.

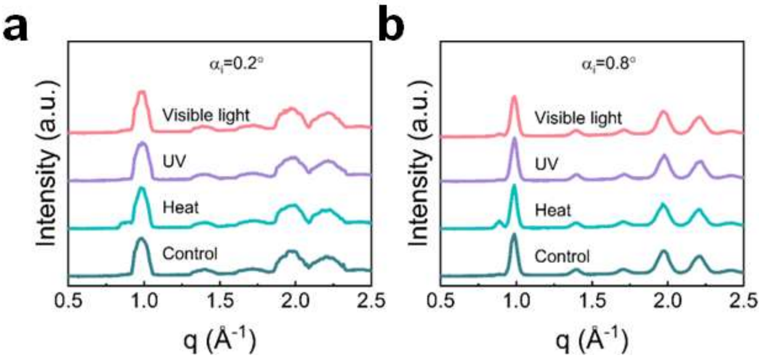

Figure S21. Pseudo-XRD data extracted from the 2D GIWAXS data by radial cake cuts measured after heating (150 °C), UV, and visible light exposure with incident angle (a) $\alpha_i = 0.2°$ and (b) $\alpha_i = 0.8°$. The exposure time for all treatments was 60 min.

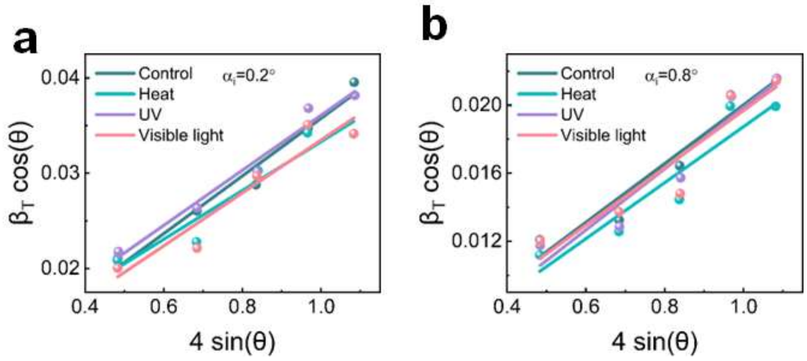

Figure S22. Calculation of the residual microstrain distribution of CsMAFA films under different conditions of exposure with incident angle (a) $\alpha_i = 0.2°$ and (b) $\alpha_i = 0.8°$.

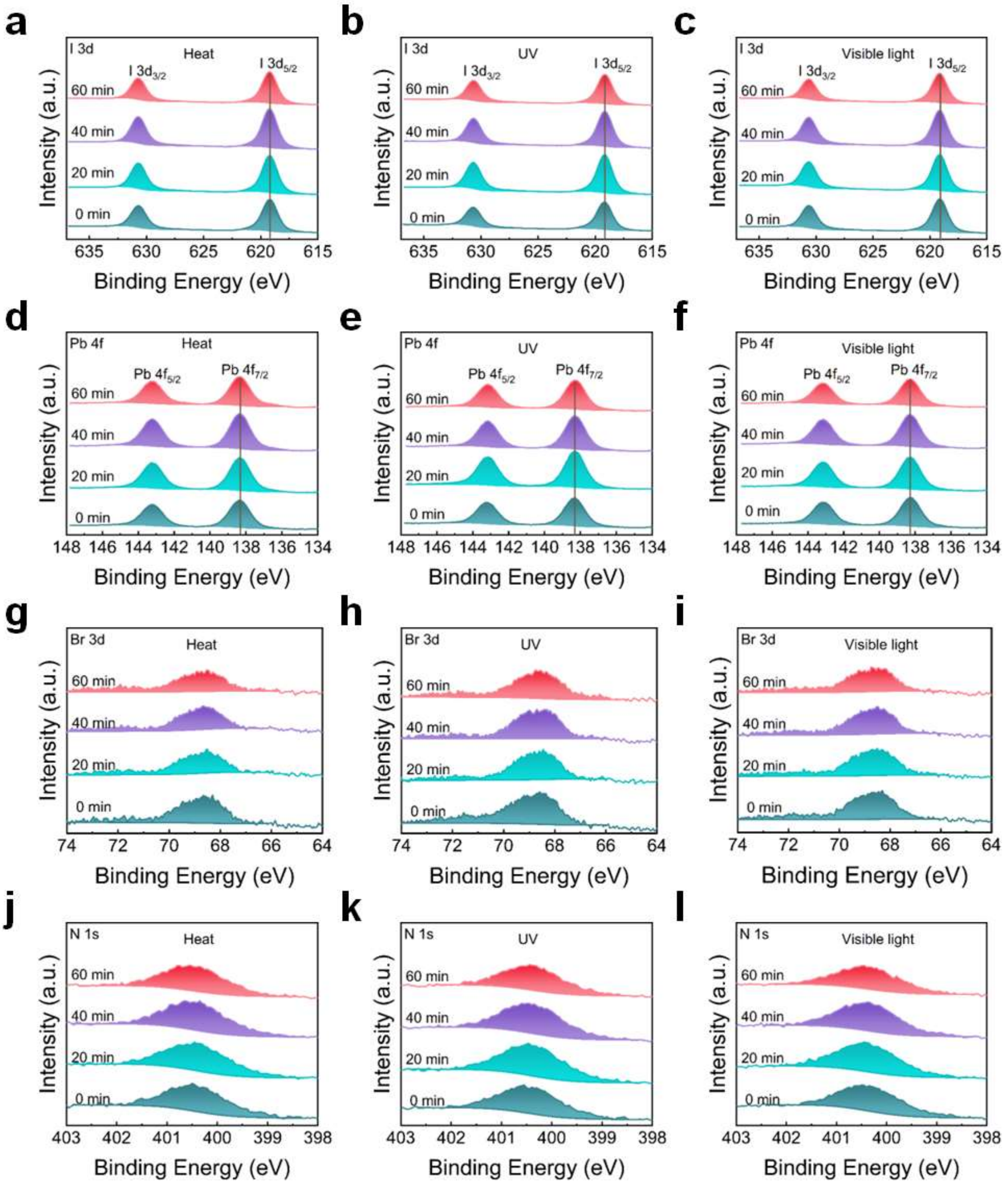

Figure S23. Time-dependent XPS core level spectra of I 3*d* exposed under different conditions of (a) heat at 150 °C, (b) UV, and (c) visible (1 Sun) light. Time-dependent XPS core level spectra of Pb 4*f* exposed under different exposure conditions of (d) heat at 150 °C, (e) UV, and (f) visible (1 Sun) light. Time-dependent XPS Br 3*d* core level spectra of CsMAFA perovskite films exposed to different conditions of (g) heating at 150 °C, (h) UV, and (i) visible light (1 Sun). Time-dependent XPS N 1*s* core level

spectra of CsMAFA perovskite films exposed to different conditions of (j) heat at 150 °C, (k) UV, and (l) visible light (1 Sun).

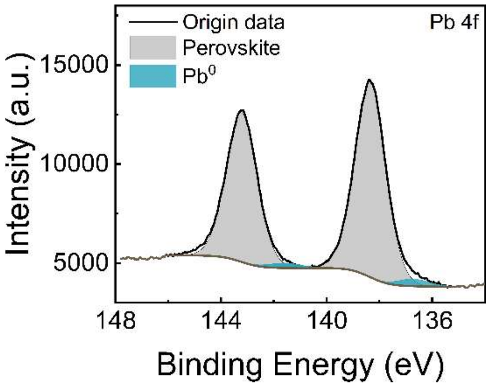

Figure S24. Pb 4*f* core level XPS spectra of CsMAFA perovskite films with fitted components indicated by color.

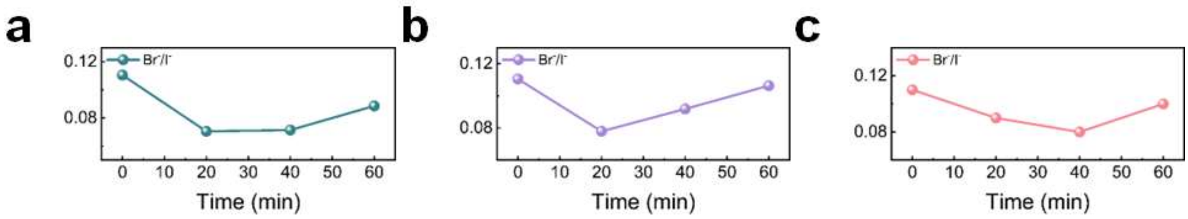

Figure S25. Changes of the I^- /Br^{-} $^{+}$ ratios as a function of exposure time under conditions of (a) heat, (b) UV, and (c) visible (1 Sun) light.

Table S1. Photovoltaic parameters of PSCs based on the ITO/SnO_2/CsMAFA/spiro-OMeTAD/Au device structure.

Devices	J_{sc} (mA cm^{-2})	V_{OC} (V)	FF	PCE (%)
1	20.28	1.16	0.78	18.41
2	20.91	1.15	0.77	18.47
3	20.91	1.16	0.77	18.54
4	20.52	1.15	0.78	18.42
5	21.04	1.16	0.78	19.02
6	21.07	1.16	0.77	18.93
7	21.10	1.13	0.80	18.93
8	20.91	1.13	0.79	18.64
9	21.55	1.15	0.77	19.15
10	22.29	1.14	0.73	18.71
11	21.40	1.17	0.77	19.27
12	22.18	1.17	0.74	19.15
13	22.22	1.16	0.78	20.10
14	20.64	1.16	0.78	18.52
15	21.46	1.15	0.77	19.13
16	21.58	1.15	0.75	18.53
17	21.64	1.15	0.79	19.60
18	21.33	1.14	0.77	18.73

19	21.51	1.16	0.75	18.83
20	21.51	1.16	0.79	19.66
20	21.51	1.16	0.79	19.66
21	20.39	1.16	0.78	18.34
22	21.27	1.15	0.77	18.71
23	21.28	1.16	0.78	19.28
24	20.46	1.15	0.78	18.48
25	22.65	1.12	0.76	19.14
26	22.91	1.15	0.74	19.52
27	22.78	1.15	0.78	20.47
28	22.87	1.15	0.77	20.45
29	21.11	1.14	0.77	18.48
30	22.87	1.15	0.73	19.25
31	22.74	1.16	0.75	19.78
32	21.30	1.15	0.78	19.18
33	21.18	1.16	0.77	19.00
34	20.96	1.15	0.78	18.79
35	22.18	1.10	0.79	19.29
Average	21.51 ± 0.77	1.15 ± 0.01	0.77 ± 0.02	19.06 ± 0.56
Champion (Forward)	22.76	1.15	0.75	19.50
Champion (Reverse)	22.78	1.15	0.78	20.47

Table S2. Microtrains in CsMAFA perovskite films heated for different times, calculated from GIWAXS data measured at $\alpha_i = 0.4°$.

Heating time (min)	0	10	20	40	60
Microstrain	0.020 ± 0.002	0.020 ± 0.002	0.021 ± 0.002	0.018 ± 0.001	0.019 ± 0.001

Table S3. Fit parameters extracted from PL spectra of CsMAFA films heated at 150 °C for different times.

Heating time (min)	0	20	40	60
Peak position (nm)	766.38 ± 0.04	767.59 ± 0.05	768.81 ± 0.04	770.20 ± 0.03

Table S4. Microtrains in CsMAFA perovskite films probed at different depths by variation of the incident angle α_i and calculated from GIWAXS data. Measurements were performed following exposure to different conditions for 60 min, as indicated in the table.

α_i (°)	0.2	0.4	0.	0.8
Control	0.030 ± 0.003	0.024 ± 0.002	0.020 ± 0.004	0.017 ± 0.003
85 °C	0.028 ± 0.019	0.024 ± 0.007	0.022 ± 0.002	0.021 ± 0.001
100 °C	0.027 ± 0.003	0.027 ± 0.002	0.021 ± 0.006	0.019 ± 0.002
150 °C	0.026 ± 0.004	0.022 ± 0.008	0.021 ± 0.007	0.016 ± 0.003
UV	0.029 ± 0.003	0.025 ± 0.004	0.020 ± 0.005	0.018 ± 0.003
Light	0.028 ± 0.005	0.024 ± 0.002	0.020 ± 0.001	0.017 ± 0.004

Table S5. Fit parameters extracted from PL spectra of CsMAFA films heated for 60 min at different temperatures.

Heating temperature (°C)	Control	85	100	150
Peak position (nm)	766.50 ± 0.03	765.54 ± 0.02	766.98 ± 0.03	770.19 ± 0.02

Table S6. Fit parameters extracted from PL spectra of CsMAFA films immersed under UV light for different times.

UV illumination (min)	0	20	40	60
Peak position (nm)	765.54 ± 0.04	765.59 ± 0.05	765.99 ± 0.04	767.41 ± 0.03

Table S7. Fit parameters extracted from PL spectra of CsMAFA films immersed under visible light for different times.

Visible light illumination (min)	0	20	40	60
Peak position (nm)	766.20 ± 0.04	766.11 ± 0.03	766.03 ± 0.03	766.23 ± 0.03

Table S8. Binding energies of Cs 3*d*, I 3*d*, O 1*s*, N 1*s*, Pb 4*f*, and Br 3*d* core level XPS spectra, as well as calculated surface compositions, obtained via fits of XPS spectra from perovskite films on Si for different heating times with a constant heating temperature of 150 °C.

			Composition (at. %)			
Name	Component	BE (eV)	0 min	20 min	40 min	60 min
Cs 3*d*	Cs^+	724.8	< 1	< 1	< 1	< 1
I $3d_{5/2}$	I^-	619.2	55	57	57	55
I $3d_{5/2}$	I^{3-}	617.5	3	4	4	3
N 1*s*	FA^+	400.4	14	13	13	13
Pb $4f_{7/2}$	Pb^{2+}	138.3	20	21	21	21
Pb $4f_{7/2}$	Pb^0	136.7	< 1	1	1	2
Br $3d_{5/2}$	Br^-	68.4	5	4	4	4
Br $3d_{5/2}$	C-Br	71.5	1	< 1	< 1	1

Table S9. Ratios for specific species, obtained from XPS spectra of perovskite films annealed for different times at 150 °C.

	I^-/Pb^{2+}	Br^-/Pb^{2+}	Br^-/I^-	$(I^-+Br^-)/Pb^{2+}$	$N(FA)/Pb^{2+}$
0 min	2.8	0.3	0.1	3.1	0.7
20 min	2.7	0.2	0.1	2.9	0.6
40 min	2.7	0.2	0.1	2.9	0.6
60 min	2.6	0.2	0.1	2.8	0.6

Table S10. Binding energies of Cs 3*d*, I 3*d*, O 1*s*, N 1*s*, Pb 4*f*, and Br 3*d* core level XPS spectra, as well as associated surface compositions, obtained via fits of XPS spectra from perovskite films on Si for different UV irradiation times.

			Composition (% at)			
Name	Component	BE (eV)	0 min	20 min	40 min	60 min
Cs 3*d*	Cs^+	724.8	< 1	< 1	< 1	< 1
I $3d_{5/2}$	I^-	619.2	55	56	56	55
I $3d_{5/2}$	I^{3-}	617.4	3	3	3	3
N 1*s*	FA^+	400.42	14	14	14	14
Pb $4f_{7/2}$	Pb^{2+}	138.33	20	20	20	20
Pb $4f_{7/2}$	Pb^0	136.64	< 1	< 1	< 1	< 1
Br $3d_{5/2}$	Br^-	68.41	5	4	5	5
Br $3d_{5/2}$	C-Br	71.45	1	< 1	< 1	< 1

Table S11. Ratios for specific species, obtained from XPS spectra of perovskite films exposed to UV for different times.

	I^-/Pb^{2+}	Br^-/Pb^{2+}	Br^-/I^-	$(I^-+Br^-)/Pb^{2+}$	$N(FA)/Pb^{2+}$
0 min	2.8	0.3	0.1	3.1	0.7
20 min	2.9	0.2	0.1	3.1	0.7
40 min	2.9	0.2	0.1	3.1	0.7
60 min	2.7	0.3	0.1	2.9	0.7

Table S12. Binding energies of Cs 3*d*, I 3*d*, O 1*s*, N 1*s*, Pb 4*f*, and Br 3*d* core level XPS spectra, as well as associated surface compositions, obtained via fits of XPS spectra from perovskite thin films on Si after different visible light (1 Sun) illumination times.

			Composition (at. %)			
Name	Component	BE (eV)	0 min	20 min	40 min	60 min
Cs 3*d*	Cs^+	724.8	< 1	< 1	< 1	< 1
I $3d_{5/2}$	I^-	619.1	55	56	56	56
I $3d_{5/2}$	I^{3-}	617.4	3	3	3	3
N 1*s*	FA^+	400.4	14	14	14	14
Pb $4f_{7/2}$	Pb^{2+}	138.3	20	20	20	20
Pb $4f_{7/2}$	Pb^0	136.7	< 1	< 1	< 1	< 1
Br $3d_{5/2}$	Br^-	68.3	5	4	4	5
Br $3d_{5/2}$	C-Br	71.5	1	< 1	< 1	< 1

Table S13. Ratios for specific species, obtained from XPS spectra of perovskite films exposed to visible light for different times.

	I^-/Pb^{2+}	Br^-/Pb^{2+}	Br^-/I^-	$(I^-+Br^-)/Pb^{2+}$	$N(FA)/Pb^{2+}$
0 min	2.8	0.3	0.1	3.1	0.7
20 min	2.8	0.2	0.1	3.0	0.7
40 min	2.8	0.2	0.1	3.0	0.7
60 min	2.8	0.2	0.1	3.0	0.7

REFERENCES

(1) Mote, V.; Purushotham, Y.; Dole, B. Williamson-Hall analysis in estimation of lattice strain in nanometer-sized ZnO particles. *Journal of theoretical and applied physics* **2012**, *6*, 1-8.
